# The Role of Crustal Buoyancy in the Generation and Emplacement of Magmatism During Continental Collision

**DOI:** 10.1029/2019GC008590

**Published:** 2019-11-05

**Authors:** Nicholas Schliffke, Jeroen van Hunen, Valentina Magni, Mark B. Allen

**Affiliations:** ^1^ Department of Earth Sciences Durham University Durham UK; ^2^ The Centre for Earth Evolution and Dynamics University of Oslo Oslo Norway

**Keywords:** Continental Collision, Magmatism, Alps, Underplating, Subducting crust, Himalaya

## Abstract

During continental collision, considerable amounts of buoyant continental crust subduct to depth and subsequently exhume. Whether various exhumation paths contribute to contrasting styles of magmatism across modern collision zones is unclear. Here we present 2D thermomechanical models of continental collision combined with petrological databases to investigate the effect of the main contrasting buoyancy forces, in the form of continental crustal buoyancy versus oceanic slab age (i.e., its thickness). We specifically focus on the consequences for crustal exhumation mechanisms and magmatism. Results indicate that it is mainly crustal density that determines the degree of steepening of the subducting continent and separates the models' parameter space into two regimes. In the first regime, high buoyancy values (*∆ρ* > 500 kg/m^3^) steepen the slab most rapidly (to 45–58°), leading to opening of a gap in the subduction channel through which the subducted crust exhumes (“subduction channel crustal exhumation”). A shift to a second regime (“underplating”) occurs when the density contrast is reduced by 50 kg/m^3^. In this scenario, the slab steepens less (to 37–50°), forcing subducted crust to be placed below the overriding plate. Importantly, the magmatism changes in the two cases: Crustal exhumation through the subduction channel is mainly accompanied by a narrow band of mantle melts, while underplating leads to widespread melting of mixed sources. Finally, we suggest that the amount (or density) of subducted continental crust, and the resulting buoyancy forces, could contribute to contrasting collision styles and magmatism in the Alps and Himalayas/Tibet.

## Key Points

- We analyse buoyancy forces in 2D thermomechanical‐petrological models of continental collision and resulting postcollisional magmatism
- Crustal buoyancy controls whether subducting crust exhumes between plates with only mantle melts or underplates with mixed melting
- Timing and distribution of crust and melting for two endmembers fits Alps and Himalaya/Tibet

## 1 Introduction

The Alps and Tibet/Himalayas are two major active continental collision zones that are strikingly different in many respects and can be seen as end‐members in a spectrum: There are strong variations in the extent of deformation, the amount, location and exhumation mechanisms of subducted continental crust, and the distribution, source, and degree of magmatism. However, the reasons why the two collision zones are so different are still debated (e.g., Ryan & Dewey, 2019).

Capitanio and Replumaz (2013) showed how the density contrast between subducting oceanic and continental domains during collision can control ongoing subduction, and partial or complete slab breakoff. The total slab buoyancy itself is determined not only by crustal thickness and density but also by the thermal buoyancy of the entire slab (Cloos, 1993). Contrast in buoyancy forces from the continent and previously subducting oceanic slab lead to an alignment (i.e., continuous steepening) of the partially subducted continent (e.g., Cloos, 1993; Duretz et al., 2012; van Hunen & Allen, 2011). The subduction angle during the steepening process in collision zones is difficult to quantify due to a strongly time‐dependent behavior. However, processes similar to those in active subduction zones may possibly influence the dynamics: During oceanic subduction the slab dip of the downgoing plate not only is a function of slab strength as well as its buoyancy (Capitanio & Morra, 2012; Stegman et al., 2010) but also depends on other parameters such as thermal state of the overriding plate (Rodriguez‐González et al., 2012), trench rollback (Lallemand et al., 2005), or lower mantle anchoring (Guillaume et al., 2009). To understand the diversity in processes during continental collision, particularly the slab steepening process, we focus on the effects of buoyancy on collisional dynamics, exhumation and location of subducting crust, and resulting magmatism.

In collision zones, considerable amounts of felsic crust subduct to depth where it separates from the slab and exhumes (known as “relamination”; Hacker et al., 2011). Contrasting locations of exhumed subducted crust in modern collision zones indicate that exhumation processes were likely different too. A variety of pathways have been unveiled numerically: The Alps might have experienced exhumation of subducting crust along the subduction interface as channel flow (Liao et al., 2018; Maierová et al., 2018; Sizova et al., 2012), while sublithospheric crustal placement for thin overriding continents has been suggest for the India‐Eurasia collision (Maierová et al., 2018). Other relamination processes include translithospheric diapirism of continental crust (Sizova et al., 2012), or, in the Archean, a diapiric upwelling through the mantle wedge (Maunder et al., 2016). Eduction is another process exhuming subducted continental crust due to its buoyancy. It represents the reverse of subduction, that is, the continent exhumes en bloc, reversing the subduction trajectory (Andersen et al., 1991; Dixon & Farrar, 1980). The process initiates after slab breakoff at depth, which removes the slab pull and allows the continent with its buoyant crust to educt (Bottrill et al., 2014; Duretz et al., 2012). This mechanism has been proposed to explain the high‐pressure (HP)‐metamorphic Western Gneiss Complex in the Scandinavian Caledonides (Andersen et al., 1991; Brueckner & Cuthbert, 2013; Teyssier, 2011).

Subducted European crust in the western Alps has been detected by local earthquake tomography within the old subduction channel (in this work, this term is used for all regions between the two continental plates, shallow and deep), doming above presumably serpentinized mantle material (Solarino et al., 2018), and rapidly exhumed HP and ultrahigh‐pressure (UHP) rocks within the orogeny (e.g., Rubatto & Hermann, 2001). In the India‐Eurasia collision the subducted Indian crust has also been exhumed as HP and UHP rocks, but, additionally, seismic studies have imaged subducted Indian crust that partially underplated Eurasia up to 200 km north of the suture zone (Chen et al., 2010; Nábělek et al., 2009). Large volumes of underthrust Indian crust have been accreted to the overriding plate within the Himalayan fold‐and‐thrust belt (e.g., Dahlen et al., 1984; Davis et al., 1983), with estimates as little as 5‐ to 15‐km‐thick lower crust continuing to subduct, leading to an overall negatively buoyant continent (Capitanio et al., 2010) and contributing to convergence between India and Asia (Guillot et al., 2003; Patriat & Achache, 1984). For microcontinents subducting in the eastern Mediterranean (e.g., Jolivet et al., 2013), which did not cause subduction to cease, crust has been subducted to depths greater than 200 km (e.g., Parra et al., 2002) and exhumed as HP or UHP rocks within a thin extended overriding plate (Blake et al., 1981; Bonneau, 1984; Laurent et al., 2017). If small amounts of subducted crust in the eastern Mediterranean fail to cease subduction, and if thinned, subducted continental crust below the Himalayas allow subduction to continue, we infer that in the Alps more crust (per unit length of trench), or more buoyant crust, has subducted to cease the subduction process.

**Figure 1:**
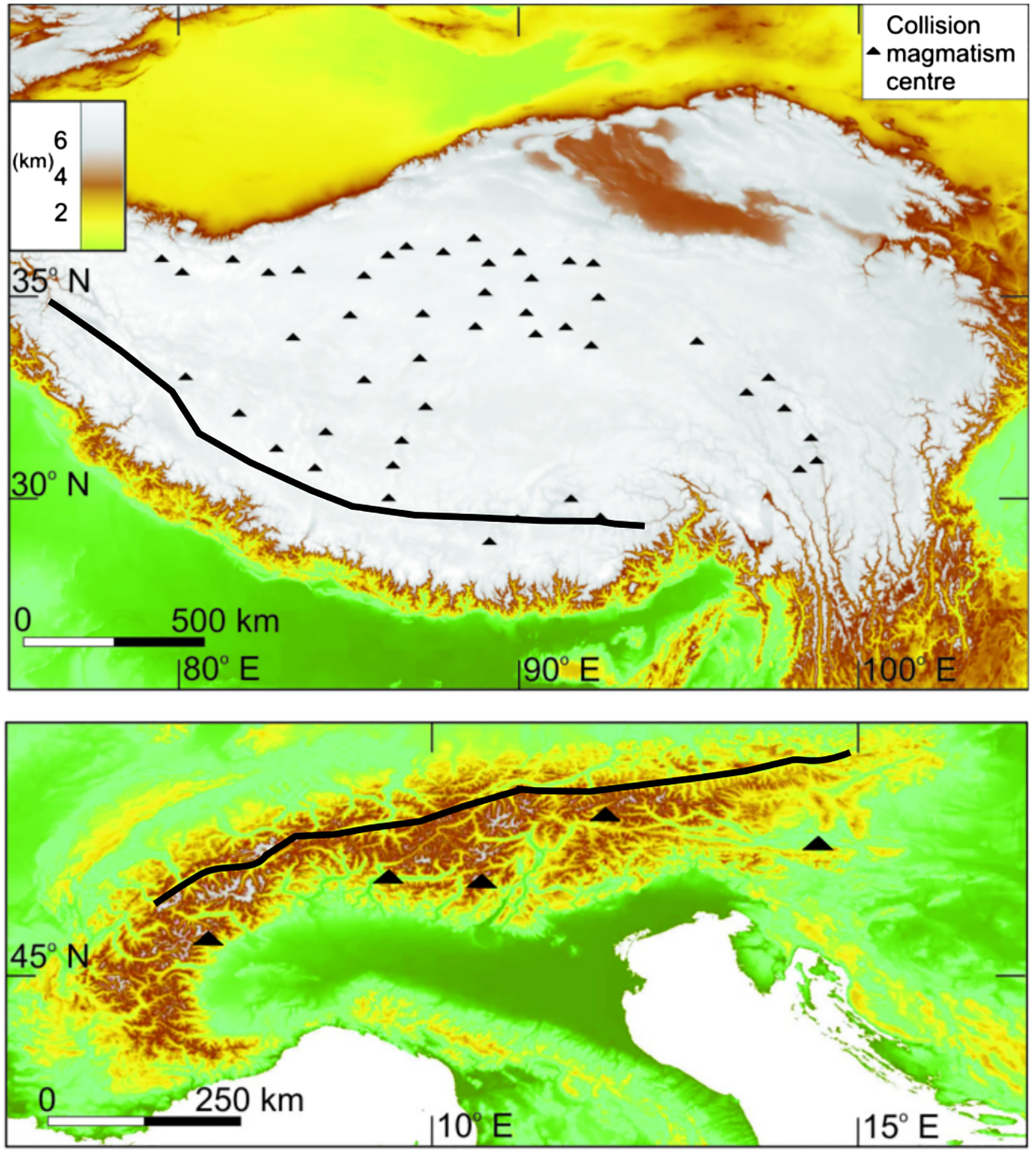
Location of the suture (black line) and distribution of syncollisional magmatism in (top) the central Tibetan Plateau (data from Chung et al., 2005) and (bottom) the Alps (data from Blanckenburg & Davies, 1995; Rosenberg, 2004). In the Tibetan Plateau, magmatism is spread widely across the plateau, while there are only a few intrusions in the Alps.

Syncollisional magmatism is a diagnostic feature of collisional dynamics through its volume, composition, and distribution in space and time. In this work, syncollisional magmatism refers to melting generated after onset of collision and during the entire collisional process that can continue for tens of millions of years (e.g., van Hunen & Allen, 2011). The Alps have very little syncollisional magmatism (e.g., Blanckenburg & Davies, 1995), which is most likely derived from a lithospheric mantle source (Rosenberg, 2004, and references therein) and could be linked to slab steepening (Ji et al., 2019). Magmatism in the India‐Eurasia collision is scattered across the central Tibetan Plateau (Figure 1). There is a complex pattern of eruption ages, but there was migration northward across central and northern Tibet, away from the suture zone (Chung et al., 2005). The age range of volcanic centres in Iran, interpreted to postdate initial Arabia‐Eurasia collision, has been used to interpret a diachronous initial collision from northwest to southeast along the collision zone (Chiu et al., 2013), but it has also been claimed that there is no clear age progression in the data (Kaislaniemi et al., 2014).

Using numerical models, Freeburn et al. (2017) show that syncollisional mantle magmatism induced by hot upwelling after slab breakoff (suggested for the Alps by Blanckenburg & Davies, 1995) is unlikely to occur for breakoff depths greater than 100 km, but melting of sediments or continental crust during the necking process can occur through heating of the mantle wedge. Andrić et al. (2018) conclude that the temporal‐spatial distribution of collision zone magmatism relates to lithospheric structure of the overriding plate. They predict melt‐induced localization of deformation and magmatism migrating along boundaries of crustal and lithospheric layers, away from the suture zone.

n this study, we illustrate how crustal buoyancy controls the angle of the partially subducted continent, which in turn determines whether the relaminating crust and syncollisional magmatism remain close to the subduction channel or extend below the overriding plate. Finally, we compare these models to Earth's active continental collision zones.

## 2 Methods

To analyze the impact of the main buoyancy forces during continental collision and track subsequent melting, a combined approach of 2D Cartesian geodynamical models with thermodynamical databases is used. To that end, we link Citcom (Moresi et al., 1996; Zhong et al., 2000), a finite element code for mantle convection, to thermodynamic databases through the software tool Perple_X (Connolly, 2005).

### 2.1 Thermomechanical Modeling

A Cartesian version of Citcom, which solves the conservation equations for mass, energy, momentum, and composition, is used. The first three equations are solved with a finite element method, while the conservation of composition uses a conservative velocity interpolation particle‐tracking technique (Wang et al., 2015).

The conservation equations assume incompressibility, absence of inertial forces in the mantle, the Boussinesq approximation (e.g., Turcotte et al., 1973), and no internal heat sources. This leads to the following equations in a nondimensional form (Normand et al., 1977; Turcotte et al., 1973):

$$\nabla \cdot \vec{u}=0$$

$$\frac{\partial T}{\partial t}+\vec{u}\cdot \vec{\nabla }T={\vec{\nabla }}^{2}T$$

$$-\vec{\nabla }p+\vec{\nabla }\cdot \eta \left[\left(\vec{\nabla }u\right)+{\left(\vec{\nabla }u\right)}^{T}\right]+\left({\mathrm{Ra}}_{T}T-{\mathrm{Ra}}_{C,i}C_{i}\right){\vec{e}}_{z}=0$$

$$\frac{{\partial C}_{i}}{\partial t}+\vec{u}\cdot \vec{\nabla }C_{i}=0$$

The thermal Rayleigh number, *Ra*_*T*_, and compositional Rayleigh number for a given composition *i*, *Ra*_*C,i*_, describe the ratio of convection driving to resisting forces and thus control the vigor. The compositional density difference between crust and mantle material *∆ρ*_*i*_ within *Ra*_*C,i*_ is one of the key parameters studied in this work. See Table 1 for all other variables.

$${\mathrm{Ra}}_{T}=\frac{\mathrm{\alpha \rho g∆T}\;h^{3}}{\kappa \eta }$$

$${\mathrm{Ra}}_{C,i}=\frac{g{\mathrm{∆\rho}}_{i}\;h^{3}}{\kappa \eta }$$

**Table 1:** Symbols, Units, and Default Model Parameters

Parameters	Symbol	Value and unit
Rheological preexponent	*A*	1 (diff. c.), 3.6 × 10^9^ (disl. c.) [Pa^−n^ s^−1^]
Activation energy	*E*	360 [kJ/mol]
Rheological power law exponent	*n*	1 (diff. c.), 3.5 (disl. c.) [−]
Lithostatic pressure	*p*_0_	[Pa]
Gas constant	*R*	8.3 [J K^−1^ mol^−1^]
Temperature	*T*	[°C]
Compositional function	*C*	[−]
Velocity	*u*	[m/s]
Vertical unit vector	$\vec{e}$_*z*_	[−]
Absolute temperature	*T*_abs_	[K]
Reference temperature	*T*_*m*_	1350 [°C]
Thermal Rayleigh number	*Ra*_*T*_	4.4 x 10^6^ [−]
Compositional Rayleigh number	*Ra*_*C*_	0.8 × 10^6^ to 2.1 × 10^7^ [−]
Gravitational acceleration	g	9.8 [m/s^2^]
Thermal expansivity	*α*	3.5 × 10^−5^ [K^−1^]
Thermal diffusivity	*κ*	10^−6^ [m^2^/s]
Compositional density contrast	*Δρ*_*c*_	280–750 [kg/m^3^]
Strain rate	$\dot{\epsilon }$	[s^−1^]
Effective viscosity	*η*_eff_	[Pa·s]
Yielding viscosity	*η*_y_	[Pa·s]
Reference viscosity	*η*_*m*_	10^20^ [Pa·s]
Viscosity difference crust‐mantle	∆*η*	0.01 [−]
Temperature drop over model	∆*T*	1350 [K]
Friction coefficient	*μ*	0.1 [−]
Reference density	*ρ*	3,300 [kg/m^3^]
Yield stress	*τ*_*y*_	[MPa]
Surface yield stress	*σ*_0_	40 [MPa]
Maximum yield stress	*σ*_max_	400 [MPa]
Model geometry		
Domain depth	*h*	660 [km]
Domain length	*l*	3,300 [km]
Overriding plate thickness	*Hop*	85 [km]
Mesh resolution		from 3.5 × 8 to 7 × 8 [km^3^]
Continental block width	—	528 [km]
Oceanic slab age	—	50, 70, 90 [Ma]
Continental crust thickness	*Hc*	40 [km]
Weak zone viscosity	*η*_weak_	10^20^ [Pa·s]

The advective part of the temperature and composition equations are transported by numerical particles (van Hunen et al., 2002). Particles also carry the information gained from the thermodynamical databases as well as assigned physical properties.

### 2.2 Model Setup

**Figure 2:**
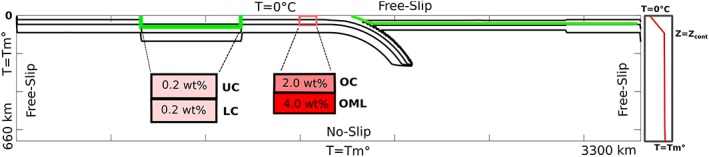
Initial model setup. A continent is embedded in the subducting lithosphere with a 40‐km‐thick buoyant crust (green contour). The overriding plate is of continental origin. Insets show the compositional layering (UC = upper continental crust, LC = lower continental crust, OC = oceanic crust, OML = oceanic mantle lithosphere) and initial hydration. Isotherms are shown every 400°C. The left thermal boundary condition (*T* = *T*_*m*_) creates a mid‐ocean ridge during model evolution.

The size of the modeled domain is 660 × 3,300 km corresponding to an aspect ratio of 1:5. The subduction process is realized by modeling two lithospheric plates converging toward each other. Subduction is purely driven by internal buoyancy forces, and immediate onset of subduction is ensured by implementing a preexisting subducting slab within the mantle (Figure 2). Gravitational forces drag the dense slab further into the mantle and create sustained subduction. A continental block (hereafter referred to as the “indentor”) is embedded in the subducting oceanic plate and is initially located ~500 km from the trench, so it does not reach the subduction zones until later in the model. The overriding plate is assumed to be entirely of continental origin. Both the overriding plate and the indentor consist of an initially 40‐km‐thick buoyant continental crust (Table 1). Thickness of the oceanic lithosphere is determined by a half‐space cooling model with an age of 70 Ma as a default value. The continental lithosphere of the indentor and the overriding plate have a linear thermal gradient from 0 °C at the surface to the mantle potential temperature (*T*_*m*_ = 1350 °C) at 120‐ and 85‐km depth, respectively. A thin weak zone with low viscosity separates the subducting and overriding plate, providing the decoupling that is required for subduction. The weak zone has a fixed viscosity of 10^20^ Pa·s and measures 7 km in thickness. It has a fixed circular shape from the trench position at surface to the base of the overriding plate at 90‐km depth. The weak zone is free to move laterally (Magni et al., 2012).

Free slip boundary conditions apply to the top and side boundaries, while the bottom boundary is no slip. To model the relatively fixed position of a large overriding plate, such as Eurasia, with respect to the deep mantle, the overriding plate is fixed to the right‐hand side model boundary by a thermal boundary condition identical to the initial depth profile. The subducting plate (consisting of the oceanic and continental parts) is free to move between a mid‐ocean ridge on the left and the trench on the right. Mantle temperature *T*_*m*_ is imposed on the left and bottom boundary, while the top boundary has *T* = 0°C. The right‐hand side boundary (i.e., the inland edge of the overriding plate) has a linear thermal gradient identical to the initial continental thermal field (i.e., to 85‐km depth), and a mantle temperature below.

The total computational domain is divided up in 600 × 62 elements in horizontal and vertical directions, respectively, containing 4 million particles. Mesh refinement is applied near the subduction zone where the high local viscosity contrast requires the finest spacing, in the upper part of the mantle (down to 178 km) and between 1,458 and 2,224 km comprising the weak zone and the mantle wedge. The typical resolution ranges from 3.5 × 8 to 7 × 12 km. A few models with an increased resolution of 800 × 124 elements are performed to assure sufficient resolving power (with the “root mean square” velocity during collision deviating by at most 1.8% between low‐ and high‐resolution models).

### 2.3 Rheology

Large viscosity contrasts at plate boundaries assist decoupling and govern slab deformation and thus subduction dynamics (Duretz & Gerya, 2013; van Hunen & Allen, 2011). The viscosity in the models is governed by diffusion creep and dislocation creep, as well as brittle yielding. A maximum viscosity *η*_max_ (set to 10^23^ Pa·s) is further applied to limit the magnitude of viscosity contrasts and substitutes for any unimplemented deformation mechanism such as Peierl's mechanism (Kameyama et al., 1999) or other low‐temperature plasticity mechanisms. Diffusion and dislocation creep follow the following flow law:

$$\eta_{\text{diff},\text{disl}}=A^{\frac{1}{n}}{\dot{\epsilon }}^{\frac{1-n}{n}}\exp\left(\frac{E}{\mathrm{nRT}}\right)$$

The power law exponent is set to *n* = 1 for diffusion creep (i.e., strain‐rate‐independent viscosity) and *n* = 3.5 for dislocation creep (Ranalli, 1995). All parameters can be found in Table 1.

To simulate the rheological differences between crust and mantle, a simple crustal weakening parameterization is applied, in which all crust is weakened in the following way:

$$∆\eta_{\text{crust}}=\eta \cdot ∆\eta^{C}$$

where *η* is the viscosity after application of the above‐described rheological laws (except for yielding and before maximum viscosity), ∆*η* the maximum amount of reduction, and *C* the fraction of crustal particles in an element (i.e., between 0 and 1). A value of ∆*η* = 0.01 is used. Reducing the crustal viscosity by 2 orders of magnitude compared to the mantle is in line with typical global models (Crameri & Tackley, 2014), to an average of numerous rheologies (Maunder et al., 2016) or comparable to rheologies such as dry mantle (Hirth & Kohlstedt, 2003) and dry quartzite (Ranalli, 1995) for the relevant *p*,*T* conditions. Within the cold, brittle deformation regime, crust and mantle material have a comparable strength, so the crustal strength reduction only takes effect above ~500 °C in our models (i.e., limited to maximum viscosity at low temperatures otherwise).

A yielding mechanism is used to model brittle failure at low temperatures and pressures, reducing the material strength. The resulting effective viscosity is implemented as

$$\eta_{y}=\frac{\min\left(\sigma_{0}+\mathrm{\mu p},\sigma_{\max}\right)}{\dot{\epsilon }}$$

in which *μ* is the friction coefficient; *σ*_0_and *σ*_max_are the surface and maximum yield stress, respectively; and $\dot{\epsilon }$ is the second invariant of the strain rate. The effective viscosity is then defined as

$$\eta_{\mathrm{eff}}=\min\left(\eta_{\text{diff}},\eta_{\text{disl}},\eta_{y},\eta_{\max}\right)$$

### 2.4 Input and Output Parameters

The buoyancy contrast between the oceanic slab and subducting continent is one of the dominant controls on the collision dynamics and primarily controlled by plate age and crustal buoyancy, which are explored in this study. The first key parameter is therefore the total buoyancy of the continental crust, which is governed by its thickness and density contrast between crust and mantle Δ*ρ*. To reduce the number of model parameters, we vary crustal buoyancy through a wide variation of crust‐mantle density contrast only while keeping crustal thickness constant. The second key input parameter affecting slab buoyancy is the age of the subducting oceanic slab. A thicker slab accounts for more slab pull and rapid subduction rates. In this study, we vary *∆ρ* between 280 and 750 kg/m^3^ (Rudnick & Fountain, 1995; Rudnick & Gao, 2003) and explore three initial oceanic slab ages (50, 70, and 90 Myr).

To analyze the results quantitatively, we measure the dip of the subducting continental plate, which varies with model parameters and during model evolution. The angle of this dip is defined at the timing of the deepest point of crustal subduction, that is, when the crustal motion changes from subducting to exhuming. The exact timing is determined by using the vertical velocity of the crustal particles, that is, the moment that the average particle vertical velocity changes sign. The subduction angle at this point is computed by measuring the angle between a reference point in the center of the weak zone and the continental crustal particle farthest away from the reference point at 160‐km depth.

### 2.5 Slab Hydration and Melt Modeling

Melting in these combined geodynamical and petrological models is evaluated using thermodynamical databases. We use the software tool Perple‐X (Connolly, 2005) to calculate stable mineral assemblages under given temperature, pressure, and bulk‐water contents and for a given composition. Look‐up tables, which list the stable mineral assemblages, are created for each composition (see Bouilhol et al., 2015, for oceanic crust, depleted mantle, and primitive mantle and supporting information [Figures S5 and S6] for continental upper and lower crust) for a relevant *P*‐*T*‐H_2_O grid (cf. Freeburn et al., 2017; Magni et al., 2014). When consulting the look‐up tables, we add an adiabatic temperature gradient to the model temperature. The material type and water content are stored on the numerical particles, which ensures diffusion‐free advection.

Although the continental crust is considered homogenous for dynamical purposes (i.e., uniform density and viscosity contrast with mantle material), petrologically, it is initially divided into two 20‐km‐thick layers (upper [felsic] and lower [mafic] crust) and has a common initial water content of 0.2 wt% (cf. Freeburn et al., 2017). In oceanic lithosphere, the 7‐km‐thick crust overlies a further 7‐km‐thick hydrated oceanic mantle lithosphere (see Figure 2). Each component is assigned an initial water content of 2 and 4 wt% for the oceanic crust and hydrated mantle lithosphere, respectively (Bouilhol et al., 2015; Faccenda, 2014; Freeburn et al., 2017).

If free excess water appears due to dehydration reactions, it is redistributed using the method described by Magni et al. (2014). It migrates vertically upward until it is able to form new hydrous minerals, it triggers melting, or until it reaches the surface. This migration is modeled to occur instantaneously; this approach makes the assumption that the water migration is much faster than solid flow. In this study we focus on the initial appearance of melt, and therefore, any occurring melt is not extracted, and no melt migration is modeled. We do not use these models to trace the total melt volume but merely to constrain the presence or absence of melt and its source. To investigate the primary dynamical influence of continental buoyancy and slab pull during continental collision and crustal detachment, we do not consider the impact of chemical composition, water content, or degree of melting on the viscosity, nor do we take latent heat into account.

## 3 Results

### 3.1 Model Evolution

By varying the crustal density as well as the oceanic slab age, we find three main types of behavior that differ in collision dynamics, final emplacement of subducted continental crust, and their magmatism. We first describe the model dynamic evolution before comparing the model with magmatic consequences.

#### 3.1.1 Subduction Channel Crustal Exhumation

**Figure 3:**
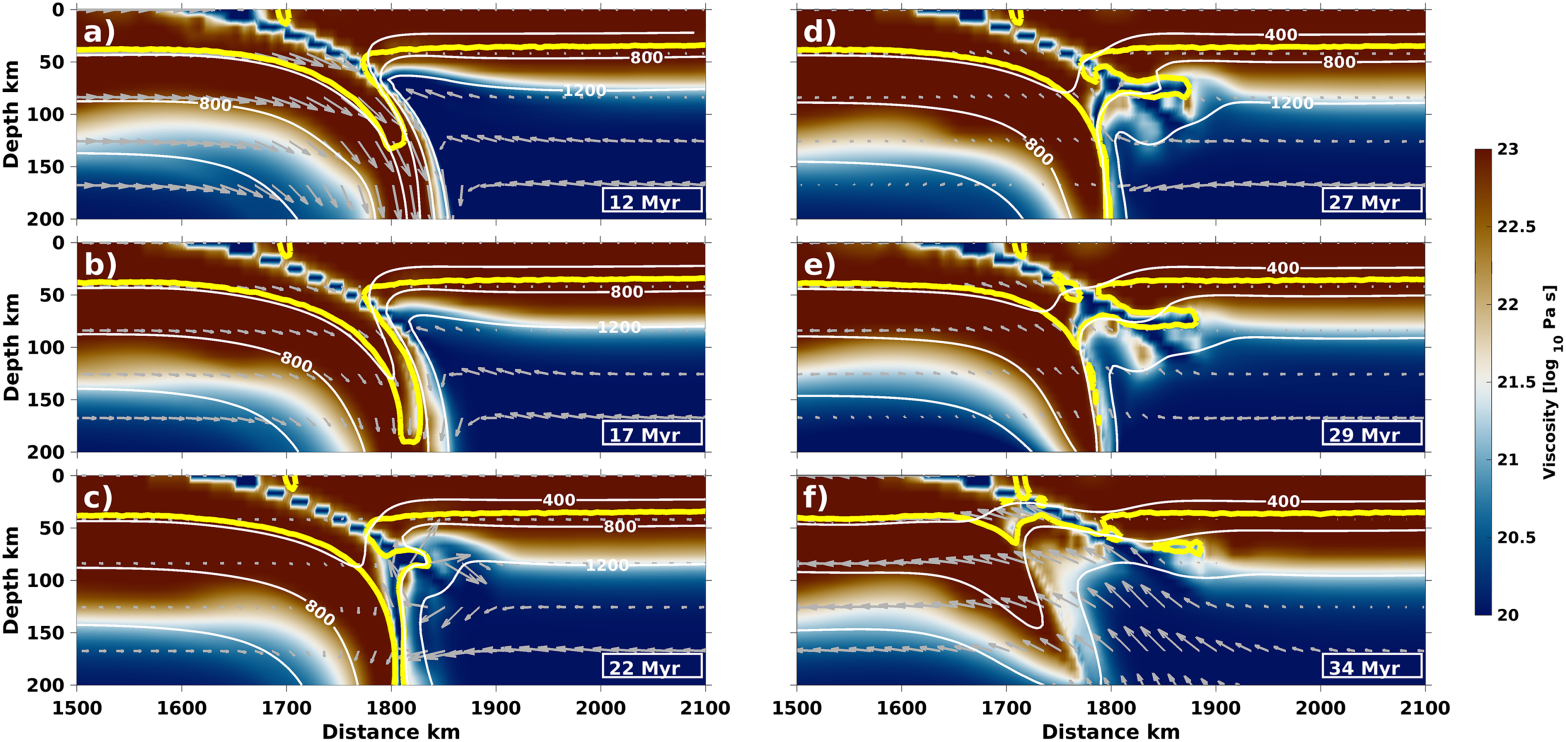
Viscosity (colors), temperature (white contours, in degrees Celsius), and velocities (gray arrows) of a characteristic model with subduction channel crustal exhumation (70‐Myr slab and ∆*ρ* = 709 kg/m^*3*^). Yellow contour outlines the continental crust. The subducting oceanic slab pulls the continent down to depths of 200 km, where the crust detaches from the continental lithosphere and flows upward into the channel as an arc‐shaped upwelling. All subducted crust ends up at shallow depths, where it causes the whole continent to educt out of the channel.

One observed regime of continental collision dynamics is referred to as “subduction channel crustal exhumation.” During initial stages of this model, oceanic subduction is initiated by the plate's negative buoyancy and slab pull, resulting in rollback at the surface. Once the continent reaches the trench and partially subducts, trench rollback stops, and convergence slows down (Figure 3a).

The continental crust enters the subduction zone and subducts to a maximum depth of up to 200–250 km (Figures 3a–3c). Opposing forces from the buoyant continental crust and the negatively buoyant oceanic slab lead to a continuous steepening of the partially subducted continent. Within ~6 Myr of entering the mantle wedge, high temperatures reduce the crustal viscosity and therefore coupling to the intrinsically stronger lithosphere. The crust detaches from continental lithosphere, flows upward (at a rate of ~5 mm/year), and forces its way into the subduction channel (Figures 3e and 3f). In the subduction channel, crust forms an arcuate upwelling and causes extension between the subducting and overriding plates. During upwelling, minor amounts (<5%) of continental crust remain attached to along the subducting continental lithosphere within the channel, as well as at the base of overriding lithosphere (e.g., Figure 3f). Exhumation of the crust in the subduction channel leads to a flow of hotter mantle material into the wedge and partially into the subduction channel (cf. isotherms in Figure 3f). Simultaneous to heating of the continental crust, necking starts near the continent‐ocean boundary and eventually leads to breakoff of previously attached oceanic lithosphere.

#### 3.1.2 Underplating

Another observed dynamic regime is referred to as “underplating.” Until onset of collision, the dynamics of subduction channel crustal exhumation and underplating are identical (i.e., subduction of the oceanic lithosphere and partial subduction of the downgoing continent).

**Figure 4:**
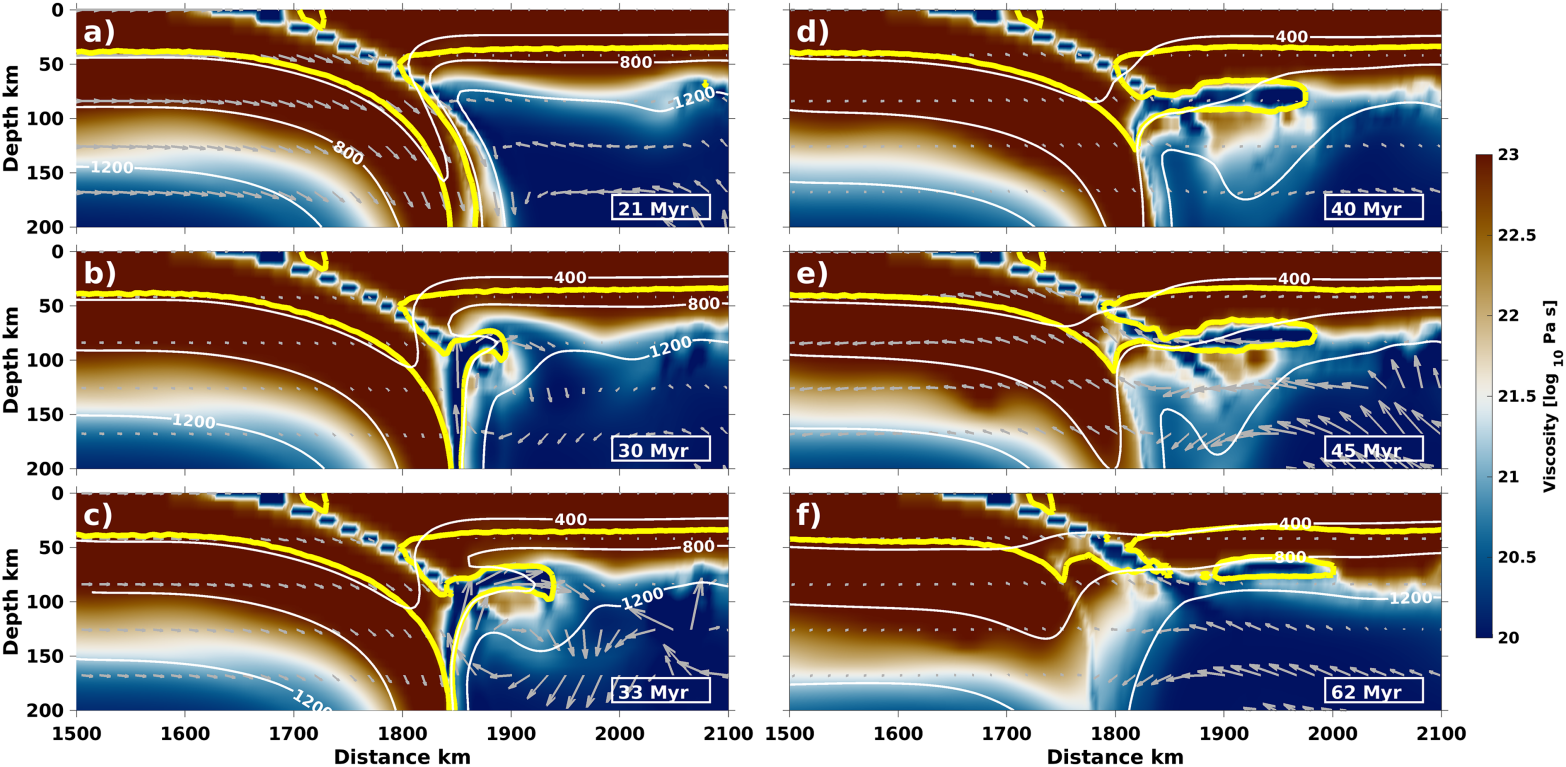
Viscosity (colors), temperature (white contours), and velocities (gray arrows) of a characteristic model with underplating (70 Myr and ∆*ρ* = 496 kg/m^*3*^). Yellow contour outlines the continental crust. The subducting oceanic slab pulls the continent down into the mantle. Before underplating, the detaching continental crust forms a plume‐like structure that gets emplaced at the base of the overriding lithosphere. This causes less crust to flow into the subduction channel.

Continental crust subducts deeper for the underplating cases than for the subduction channel crustal exhumation cases, to maximum depths of 250–300 km (Figures 4a–4c). In the process, the subducting continent also steepens but to a lesser extent, so that, at the moment of crustal detachment, a lower dip angle of the partially subducted continent triggers a crustal upwelling to spread into a wider, plume‐like structure (Figures 4b and 4c). The upwelling reaches the base of the overriding plate, away from the subduction channel (Figures 4c–4e). During this period, convergence between the continents continues (e.g., velocities of the subducting continent in Figure 4c). After the emplacement of most of the subducting continental crust at the base of the overriding plate, the remaining crust in the subduction channel (as well as the subducting continent) begins to exhume (Figures 4e and 4f). This, however, happens to a far lesser extent than during subduction channel crustal exhumation. Nevertheless, exhumation drives hot mantle material into the mantle wedge where it replaces the exhumed crust, causing an overall rise of temperature in the wedge, at the base of the overriding plate and in the underplated subducted crust (cf. rising of 800 °C contour in Figure 4f).

#### 3.1.3 Whole Crustal Subduction

The third regime is whole crustal subduction. In this regime, the continental crust completely subducts into the mantle, where it partly underplates and is partly entrained deeper (Figure S4). The entrance of continental crust at the trench leads to a reduced convergence velocity and a steeper subduction angle, but it does not cease subduction.

We mention this regime to show the end‐member at the lower bounds of the parameter range. However, as “whole crustal subduction” does not involve breakoff and subduction cessation, we do not discuss this case any further.

### 3.2 Model Comparison

**Figure 5:**
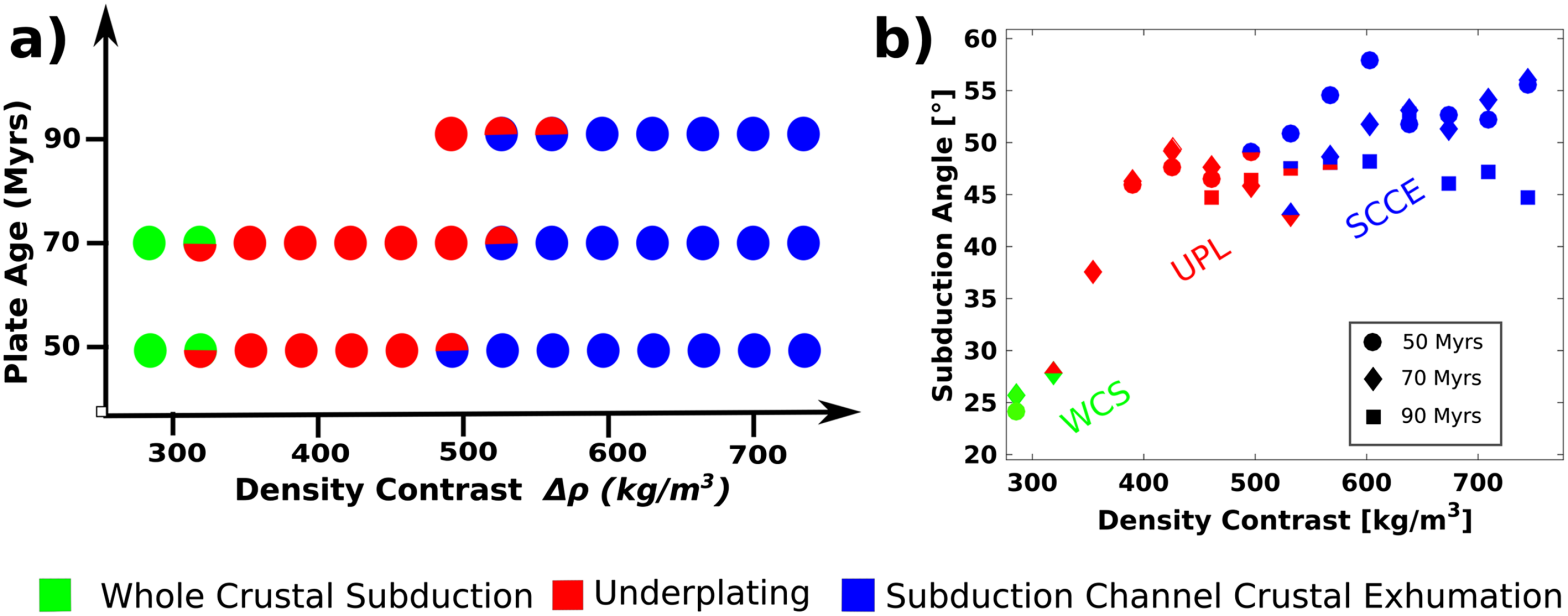
(a) Parametric study of slab crust dynamics as a function of density contrast between crust and mantle and oceanic plate age, with density contrast being the main control: low density contrasts lead to subduction of the whole continental crust (green), intermediate density contrasts trigger underplating (red), and high density contrast causes the continental crust to be emplaced in the subduction channel (blue). Bicolored symbols are transitional regimes (see Figure S3). (b) Dip angle of the partially subducted continent immediately before breakoff. The regime is a function of the density contrast between crust and mantle for all runs. Steeper angles of the continental crust increase the likelihood of exhumation in the subduction channel (SCCE), whereas shallower subduction angles account for the formation of plume‐like structures and underplating (UPL). The angle for whole crust subduction (WCS) is close to oceanic subduction. The angle of WCS is estimated during the onset of crustal detachment.

**Figure 6:**
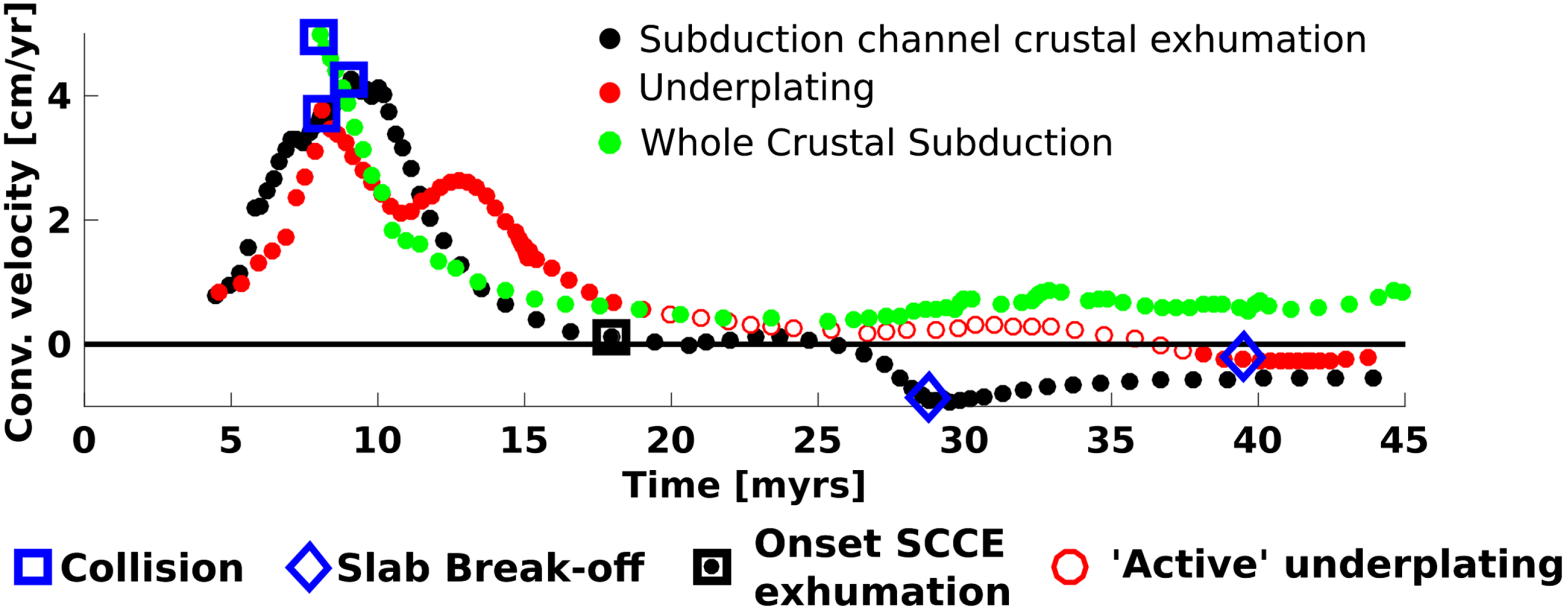
Convergence rates and timing of collision, exhumation and slab breakoff in the three presented models (all with a 70‐Myr slab and ∆*ρ* = 280 kg/m^*3*^ [whole crustal subduction], ∆*ρ* = 496 kg/m^*3*^ [underplating], and ∆*ρ* = 709 kg/m^*3*^ [subduction channel crustal exhumation, SCCE]). While convergence stops rapidly for subduction channel crustal exhumation, it continues during the actual underplating phase (but ceases before breakoff) and during entire whole crustal subduction. No data before collision in the whole crustal subduction are plotted, because the convergence is significantly higher than that in the other two models.

Figure 5a summarizes the dynamics of the entire parameter range in this study. Whole crustal subduction occurs for low density contrasts between crust and mantle (*∆ρ* = 280 kg/m^3^, Figures 6 and S4), underplating at intermediate density contrasts (*∆ρ* ≈ 350–470 kg/m^3^, *∆ρ* ≈ 350–480 kg/m^3^, and *∆ρ* ≈ 480 kg/m^3^ for slab ages of 50, 70, and 90 Ma, respectively) and subduction channel crustal exhumation at upper bounds of the parameter range *∆ρ* > 500 kg/m^3^, *∆ρ* > 550 kg/m^3^, and *∆ρ* > 580 kg/m^3^ for oceanic plate ages of 50, 70, and 90 Myr, respectively. Models with a 90‐Ma‐old oceanic lithosphere and lower density contrasts are not included because those slabs start to fold in the deep mantle. This results in completely different dynamics as also shown in Di Giuseppe et al. (2008) and so not comparable to other model runs.

Between the defined dynamic regimes there is a narrow parameter range with transitional behavior (Figures 5 and S3), in which characteristics of both subduction channel crustal exhumation and underplating are observed. For example, at a density contrast of 530 kg/m^3^ and an oceanic plate age of 70 Myr, the continental crust partially underplates and partially flows back into the subduction channel. We define the threshold of underplating when more than 30% of the detached and exhumed continental crust underplates.

Changing dynamics in the models with slab breakoff are linked to the subduction angle during collision (Figure 5b): High crustal buoyancy quickly ceases subduction and causes the slab to steepen most rapidly due to opposing forces from the buoyant continental crust and negatively buoyant oceanic slab below. By the time crustal detachment occurs, the partially subducted continent has steepened to different angles in the various regimes. For subduction channel crustal exhumation, the angle of the partially subducted crust is steeper (~45–58°, Figure 5b) than before underplating (~37–50°), which is a central result in this study: for a steep slab, crust tends to exhume vertically along the slab (Figure 3), while less steep slabs provide the possibility of a plume‐like structure (Figure 4). The angle of the subducted continent during whole crustal subduction is clearly lowest because of ongoing convergence.

The low density contrasts between crust and mantle generate little crustal buoyancy in whole crustal subduction, hence an overall negative continental buoyancy, ongoing “slab pull,” and convergence (Figure 6). While high crustal buoyancy ceases subduction channel crustal exhumation models rapidly and causes early slab breakoff, there still is reduced convergence during the underplating phase. Ongoing convergence during underplating is due to the inland directed motion of the underplating crust away from the trench. Only when underplating is completed and slab breakoff occurs, convergence ceases completely. Faster reduction of convergence rates in subduction channel crustal exhumation models also limits the time that crust spends in the mantle wedge and associated thermal weakening, compared to underplating models. This implies that crust will be weakened more with onset of exhumation in the underplating models.

Older oceanic plates do tend to shift the limits separating the regimes slightly to higher density contrasts (Figure 5a). This is explained by increased rigidity of older subducting slabs, leading to a slower steepening of those slabs during continental collision. For the same reason, older slabs experience lower subduction angles for the same crustal density values (see “outliers” at 90 Myr and *∆ρ* = 680–720 kg/m^3^ in Figure 5b).

Variation of crustal buoyancy between models is achieved by varying the density contrast between continental crust and the ambient mantle. But a similar effect can be achieved using different crustal thicknesses. We tested the difference in dynamics between both ways to vary buoyancy. Starting from the representative runs of the subduction channel crustal exhumation and underplating models, we halved the crustal thickness and doubled the crustal buoyancy to achieve similar total buoyancy values. Both models exhibit the same dynamical characteristics as the “original” models (see supporting information Figures S1 and S2). The total amount of exhumation or underplating crust is less, however, as overall crustal volume of the initial crust is lower.

### 3.3 Crustal and Mantle melting

Partial melting of continental crust and asthenospheric and lithospheric mantle occurs during both underplating and subduction channel crustal exhumation and will be used here as a diagnostic tool for the identification of collision zone dynamics.

**Figure 7:**
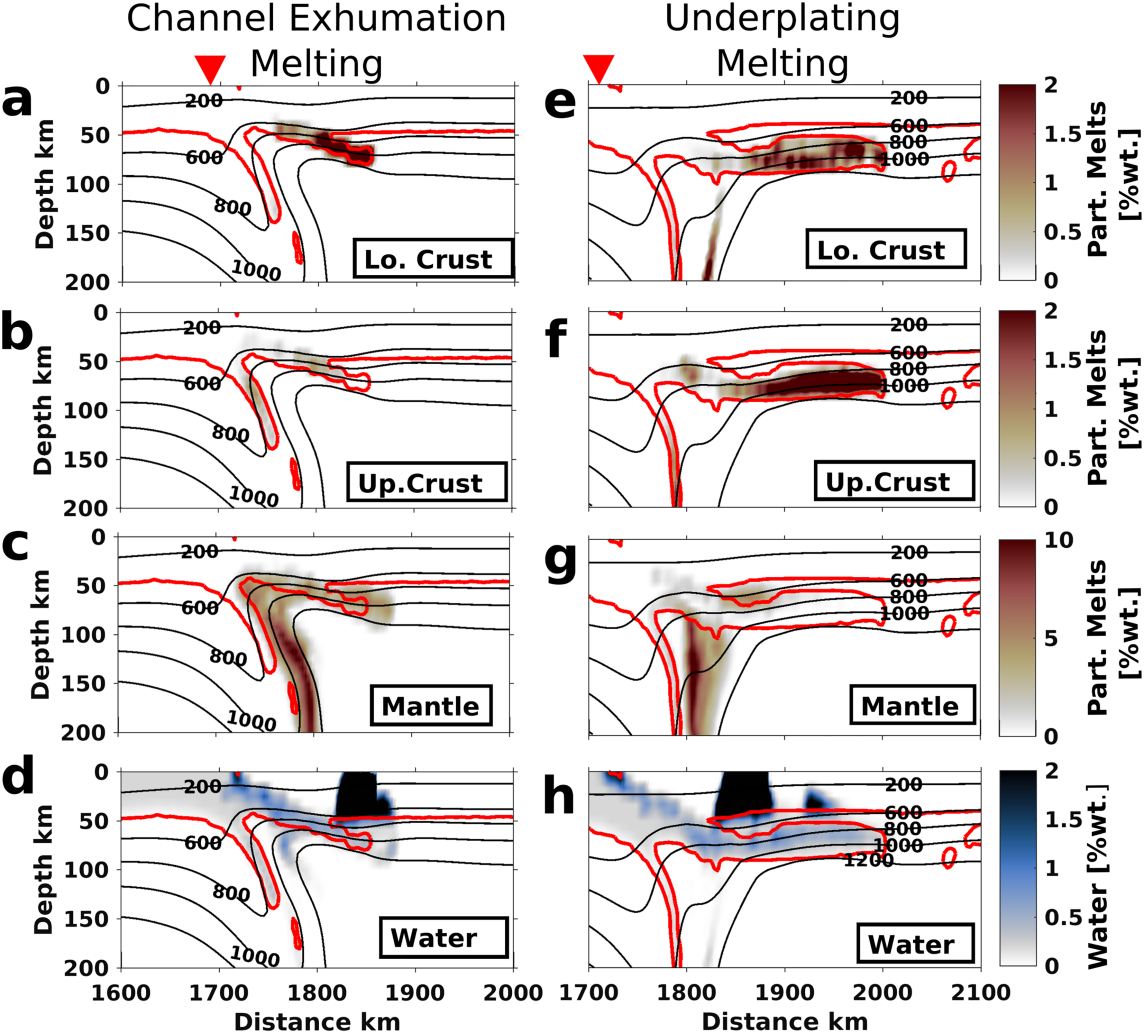
Characteristic stages of (a–d) subduction channel crustal exhumation (70‐Myr plate, ∆*ρ* = 709 kg/m^*3*^ at *t* = 34 Myr; cf. Figure 3f) and (e–h) underplating (70‐Myr plate, ∆*ρ* = 496 kg/m^*3*^ at *t* = 40 Myr; cf. Figure 4d) showing the amounts of lower crustal, upper crustal, and mantle melts. The combined continental upper and lower crust (partly mixed during the relamination process) is outlined in red, and the suture position at surface is marked with a red triangle. During subduction channel crustal exhumation, crustal melting is limited and occurs mostly where remnants of continental crust remain attached to the overriding plate. Exhumation results in mantle decompression melting within the channel. More crust partially melts during underplating due to its more extensive exposure to the hot mantle wedge. Mantle melts are present but less affected by the flow of crustal material during underplating. Note that the total degree of melting is higher for mantle material in both cases.

During subduction cessation and the resulting necking process, the final stage of oceanic crustal dehydration occurs and water percolates upward. This free water is redistributed and absorbed in three different areas: (1) as mineral‐bound water in the subducting continental crust, (2) in primitive melts in the mantle wedge, and (3) in the overriding lithosphere, partially in melts and partially as mineral‐bound water (Figure 7). For details of the initial oceanic subduction phase (i.e., dehydration of the slab and mantle wedge melting), please refer to Freeburn et al. (2017).

**Figure 8:**
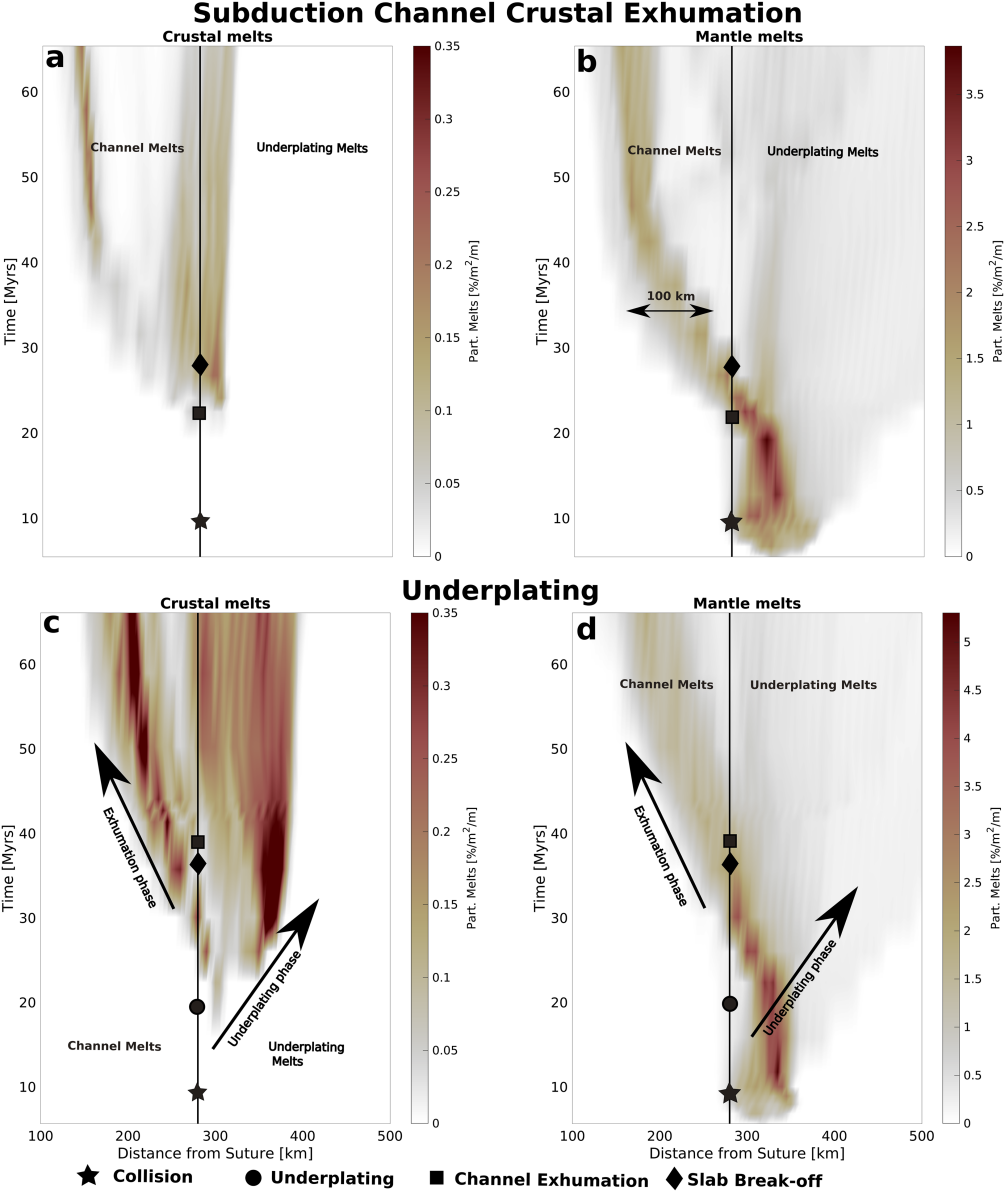
Temporal evolution of the distribution of crustal and mantle melts relative to the trench position at the surface for (a, b) subduction channel crustal exhumation (70‐Myr‐old slab and ∆*ρ* = 709 kg/m^*3*^) and (c, d) underplating (70‐Myr‐old slab and ∆*ρ* = 496 kg/m^*3*^). Underplated and channel melts are roughly separated by the black line. In the first case, crustal melting is very scarce, but mantle melting within the subduction channel extends ~100 km laterally and progressively moves toward the suture. For underplating, crustal melts migrate away from the suture until it has reached the final position (~35 Myr and ~400 km). The exhumation within the subduction channel (after ~38 Myr) increases temperature within the wedge and leads to a second phase of crustal melting. Mantle melting not only is less affected by the dynamics but is also present during most time steps. See Figures S7–S10 for detailed melt evolution.

The different dynamics after onset of exhumation in underplating and subduction channel crustal exhumation regimes result in contrasting composition and distribution of melts, both spatially (Figure 7) and temporally (Figure 8). For more detailed figures, please see Figures S7–S10.

#### 3.3.1 Melting During Subduction Channel Crustal Exhumation

Most melting typically takes place in the (predominantly asthenospheric) mantle in a spread of ~100 km laterally within the subduction channel (Figure 7c) where hot asthenosphere is rising and melting is supported by hydration (Figure 7d). An overall increase of temperature during exhumation partially melts small volumes of continental crust in areas where it was attached to the overriding plate during exhumation (e.g., lower crustal melts at *x* = 1,850 km; Figure 7b).

Figures 8a and 8b show the temporal and spatial evolution of both crustal and mantle melting, respectively, during subduction channel crustal exhumation. During subduction cessation and the onset of collision, only mantle melting within the mantle wedge occurs. Crustal exhumation (from ~20 Myr) causes the melt pattern to change: The distribution of magmatism seen in Figure 7 is formed and remains stable for a long time interval (20–40 Myr), with little crustal and mantle melting occurring within the subduction channel. In this period, the dominant area of mantle melting (and to a lesser degree crustal melting) shifts progressively closer to the suture in one continuous phase, while the ~100‐km lateral extent of mantle melting remains stable throughout this period. The degree of mantle melting is significantly higher than the amount of crustal melting (cf. color scales in Figures 8a and 8b).

#### 3.3.2 Melting During Underplating

For the underplating regime, the source, location, and timing of partial melting differ strongly from those of the subduction channel crustal exhumation regime (Figures 7 and 8). The characteristic stage in Figure 7 shows widespread crustal melting below the overriding plate and mantle melting centered above the remaining lithosphere below. Compared to that at subduction channel crustal exhumation, the temperature in the mantle wedge is much lower at this stage (cf. 1200 °C isotherms).

Breakoff and crustal exhumation occur significantly later in underplating models due to lower buoyancy, increasing subduction depth and period the crust is located within the mantle wedge. This causes the crustal melting in the underplating cases to start melting earlier (Figure 8), just before crustal detachment occurs at ~20 Myr. Afterward, the detached crust rises through the hot mantle wedge, thereby considerably increasing the average temperature of this crust and enabling partial melting due to decompression. During the ponding and spreading of continental crust beneath the overriding plate, the far end of the distribution of crustal magmatism migrates away from the suture zone (underplating phase ~20–35 Myr, up to 400 km; Figure 8). The final emplacement of the crust beneath the overriding plate leads to a permanent exposure of the continental crust to high temperatures, resulting in crustal melting at depths of ~90 km and temperatures of about 900 °C (Figures 7e and 7f). However, the overall temperature of the mantle wedge is decreased by the plume breaking away from the cold continental lithosphere. Once the underplated continental material has stagnated (at ~35 Myr), the remaining crust in the subduction channel exhumes and is replaced by hot mantle material, thereby increasing the temperature in the mantle wedge and the underplated material. The temperature increase causes a second phase of crustal melting (exhumation phase; Figure 8c). Throughout the model evolution, mantle melts are mostly limited to areas above the dehydrating slab (Figure 7g) and is hardly affected by the sublithospheric flow away from the suture during underplating.

In models with parameters between the two presented models of subduction channel crustal exhumation and underplating (Figure 5a), there is a gradual shift from local dominated mantle source melting for high crustal buoyancy to more widespread mixed‐source magmatism for lower crustal buoyancy. Lower crustal buoyancy values increase the amount of crustal underplating and hence also the amount of crustal melts in the hot mantle wedge as well as the maximum distance between suture and melting.

## 4 Discussion

### 4.1 Regime Evolution

The presented models show how buoyancy of continental crust determines whether crust completely subducts, migrates into the subduction channel, or partially underplates the overriding plate. During these processes, crustal melting roughly correlates with the emplacement position of the partially subducted continental crust.

The angle of the partially subducted continent governs the difference in dynamics after breakoff. In our models, this is dominantly controlled by the density contrast between crust and mantle, and, to a much lesser degree, by oceanic plate age (Figure 5b). Less crustal buoyancy accounts for a shallower angle and for crustal underplating, while high crustal buoyancy leads to a steep subduction angle and exhumation along the continental lithosphere and into the subduction channel. Thin overriding plates cause both shallow subduction angle during oceanic subduction (Rodriguez‐González et al., 2012) and underplating during continental collision (Maierová et al., 2018), suggesting that similar parameters control the angle during oceanic subduction and steepening process during collision. Although rollback can be ruled out as source of low‐angle subduction during collision (Lallemand et al., 2005), parameters such as the strength of subducting plates (Capitanio & Morra, 2012) could be crucial for the angle of continental subduction and could be further addressed in the future.

While crustal relamination by subduction channel crustal exhumation has been shown in several studies, in which the exhumation of crust within the channel leads to asthenospheric mantle being dragged upward in between the two continental plates (Duretz & Gerya, 2013; Freeburn et al., 2017; Liao et al., 2018; Maierová et al., 2018), models of underplating are much less common. Underplating including the continent's lithospheric mantle has been proposed to be caused by far‐field forcing (Chemenda et al., 2000) or as a 3D effect from either continuous retreating neighbouring slabs (Li et al., 2013; Magni et al., 2017) or partial lateral slab breakoff (Capitanio & Replumaz, 2013). Only Maierová et al. (2018) recently modeled relamination by crustal detachment from the slab and progressive sublithospheric movement for thin overriding continents. On a smaller scale, Vogt and Gerya (2014b) predict buoyant oceanic plateaus with weak crust to underplate, if sufficient slab pull remains. Generally, weak crustal rheologies are required to detach crust from the lithosphere at depth in all previous models of relamination (e.g., Duretz & Gerya, 2013; Liao et al., 2018; Maierová et al., 2018) as well as in this work where crustal viscosity is reduced by two orders of magnitude. This viscosity contrast has been used in global models (Crameri & Tackley, 2014) and is an average difference between of many crustal and mantle rheologies (Maunder et al., 2016). Weaker rheologies, such as wet quartzite, decouple the crust at far shallower depths (Andrić et al., 2018; Vogt & Gerya, 2014a) before it could reach the mantle wedge and underplate. Lower friction coefficients reducing crustal strength in the brittle domain (e.g., Turcotte & Schubert, 2014), hydration (Arcay et al., 2005), or other mechanisms to weaken the crust at shallow depths would likewise lead to a more rapid crustal separation and channel exhumation. In contrast, absence of crustal weakening causes buoyant crust to be within the hot mantle wedge for a longer period without detaching and exhume wholesale with the lithosphere after slab breakoff (Bottrill et al., 2014; van Hunen & Allen, 2011). Besides the strength of crust, phase changes causing densification could alter the model results. In particular, a subducting passive margin with a large volcanic arc would cause a larger than usual increase of density, thereby increasing convergence velocities (Afonso & Zlotnik, 2011). Depending on composition and degree of eclogitization of the (mafic) lower crust, its density could increase to just below (Austrheim, 1991; Bousquet et al., 1997) or even up to 300 kg/m^3^ above mantle densities and result in an overall negatively buoyant continent. At the depth of 250–300 km, high‐density phase changes can further increase the crustal density to above the mantle density (Afonso & Zlotnik, 2011), which would inhibit breakoff and relamination.

### 4.2 Consequences for Melting

The models in this study suggest that the distribution and source of syncollisional melting strongly depends on the buoyancy of the subducted continental crust and dynamics subsequent to breakoff. During subduction channel crustal exhumation, we expect some local (~100 km) mantle melting, without much crustal melting. In the underplating regime, melting migrates in two phases (i.e., underplating and exhumation), is widespread (up to 400 km from the suture), and shows a mix of crustal and mantle melting. A diverse nature of syncollisional melting has also been predicted in Freeburn et al. (2017): Asthenospheric mantle melting directly related upwellings after breakoff should only occur for very shallow breakoff depths (i.e., by high continental buoyancy) while postbreakoff melting can appear as mantle melts during delamination (i.e., peeling of lithosphere from the crust at the surface for models with a weak lower crust) or as crustal melting during extension. Our models also confirm the likelihood of sublithospheric movement of crust causing crustal melting migrating away from the suture, which has also been observed in other numerical models as movement along layer boundaries at either crustal or lithospheric scale (Andrić et al., 2018; Maierová et al., 2018). Most melting in our models originates at sublithospheric depths or within the subduction channel, which strongly contrasts the finding of Andrić et al. (2018) that melting occurs as shallow 50 km, weakens the overriding plate, and causes melt movements along compositional boundaries.

Neglecting latent heat in these models is a significant simplification. Taking latent heat into account would cause a temperature drop and reduction of melting, particularly in underplating models with the significantly higher melt fraction compared to that in subduction channel crustal exhumation. Latent heat is likely to be subordinate to advective heat transport in areas with flux melting such as subduction zones (Rees Jones et al., 2018) or during crystallization at crustal scale (Melekhova et al., 2013), so the neglection should not have major effects in these models. Also, the overall trend of having limited mantle magmatism within the subduction channel during subduction channel crustal exhumation, and relatively more mixed‐source magmatism during underplating of continental crust, should not be affected by neglecting latent heat.

### 4.3 Comparison to Cenozoic Collision Zones

We suggest that our models of subduction channel crustal exhumation could be applicable to the Alpine orogeny, with most subducted crust exhuming through the subduction channel as doming crust ~20 Myr after initial collision. The orogeny has large structural variations along strike, with subducted HP and UHP continental crust exhumed locally (Solarino et al., 2018; Zhao et al., 2016) and partially doming above mantle material in the western Alps (Solarino et al., 2018). Only thin slivers of accreted crust at the surface (e.g., Schmid et al., 2004) and a partially subducted European continent (Zhao et al., 2015) suggest that large parts of the subducting crust did reach mantle depths. These would generate high buoyancy values and could have caused rapid steepening of the slab and crustal exhumation between the continental plates as in our models. Slab steepening and enhanced corner flows have also been suggested to be the reason of peri‐Adriatic melting, aged ~30 Ma, in the western Alps (Ji et al., 2019), however in absence of breakoff. More synchronous mantle melting (Rosenberg, 2004, and references therein) occurred sporadically along the strike of the whole Alpine orogen, roughly parallel to the original suture and no more than 100 km perpendicular to the former trench (Blanckenburg & Davies, 1995). Formerly suggested sources of mantle melting, such as hot upwelling of asthenosphere after slab breakoff (Blanckenburg & Davies, 1995), seem not to match observations (Ji et al., 2019) or numerical models (Freeburn et al., 2017), unless breakoff depths occur at very shallow depths (<100 km). We thus suggest crustal exhumation along the subduction channel to have caused melting in the Alps.

Unlike the Alps, the India‐Eurasia collision zone has associated deformation distributed over a large area (up to ~1,000 km away from the original suture), exhibits widespread magmatism of multiple sources (Chung et al., 2005), and has shown strong ongoing convergence (Guillot et al., 2003; Patriat & Achache, 1984). Ongoing convergence could be supported by far‐field forcing (Chemenda et al., 2000) and continuous retreat of neighboring slabs (Li et al., 2013; Magni et al., 2017), or it could result from ongoing pull from a negatively buoyant continent, if only lower crust subducts (Capitanio et al., 2010). The latter would partly agree with our underplating models in that less (or lighter) crust is subducted in India‐Eurasia collision than in the Alps. Unlike those in Capitanio et al. (2010), however, our models would lead to eventual subduction cessation and slab breakoff.

Our underplating models explain a reduction of convergence rate, and also coeval placement of the subducting crust beneath the overriding lithosphere, as observed in parts of Eurasia where the subducted Indian crust has been imaged to have underplated Eurasia as far as 250 km away from the suture (Shi et al., 2015; Shi et al., 2016; Wittlinger et al., 2009). While some global tomography studies have imaged the subducting lithosphere to reach mid‐mantle depths (van der Voo et al., 1999; Replumaz et al., 2004), other studies, however, show the Indian crust to have underplated together with its continental lithosphere (Chen et al., 2010; Shi et al., 2016), which would not agree with our models and require different dynamics such as ongoing subduction of neighboring plates (Magni et al., 2017).

During the underplating phase (20–35 Myr; Figures 8c and 8d), there is a shift of melting (mainly from the subducted continental crust) up to 400 km away from the suture, which could explain a ~300 km northward shift in ~20 Ma with a large spread of melt sources in the central Tibetan Plateau (Chung et al., 2005; Guo et al., 2006). Zircons originating from the Indian crust and found in magmatic rocks close to the suture in the Himalayan belt suggest that some magmatism may well be partially derived from subducted continental crust (Bouilhol et al., 2013), consistent with the Sr‐Nd‐Pb isotope systematics of magmatism further north (Guo et al., 2006). Missing compositional variations or small‐scale processes at shallower depth (e.g., faulting and shear heating) in our models prevent predictions of melting at shallow depths, such as crustal melting due to thickening of the overriding crust during collision (e.g., Chen et al., 2018; Wang et al., 2016).

In summary, our models could explain first‐order observations, that is, the geometric crustal and magmatic structure, in the Alps and Himalaya/Tibet. The results suggest that more crust, or less dense crust, per unit trench has subducted in the Alps than during the India‐Eurasia collision, contributing to the striking differences in these Cenozoic collision zones.

## 5 Conclusions

Our models address the influence of buoyancy forces on continental collision dynamics, with a specific focus on the continental subduction angle. We show that after subduction cessation and slab breakoff, the subducted continental crust either is gathered within the subduction channel or partially underplates the overriding plate. We find that density contrast between the subducting continental crust and the lithospheric mantle has a crucial influence on these syncollisional dynamics. In particular, a higher density contrast results in high subduction angle and favors the occurrence of subduction channel crustal exhumation, whereas underplating is most likely to occur at lower density contrasts (lower subduction angle). The resulting syncollisional magmatism is predicted to be very local (~100 km from the suture) and mostly mantle derived for channel exhumation, while during underplating widespread magmatism is likely, with both crustal and mantle origins.

## Supporting information

Supporting Information S1
