# Supplementary material for: The Role of Crustal Buoyancy in the Generation and Emplacement of Magmatism During Continental Collision
Source: Geochem Geophys Geosyst. 2019 Nov 5;20(11):4693–709. doi: 10.1029/2019GC008590 (PMC6988479; doi:10.1029/2019GC008590)
Supplement: Supplementary file 1 — Supporting Information S1 [file GGGE-20-4693-s001.docx]

**Supplementary Material**


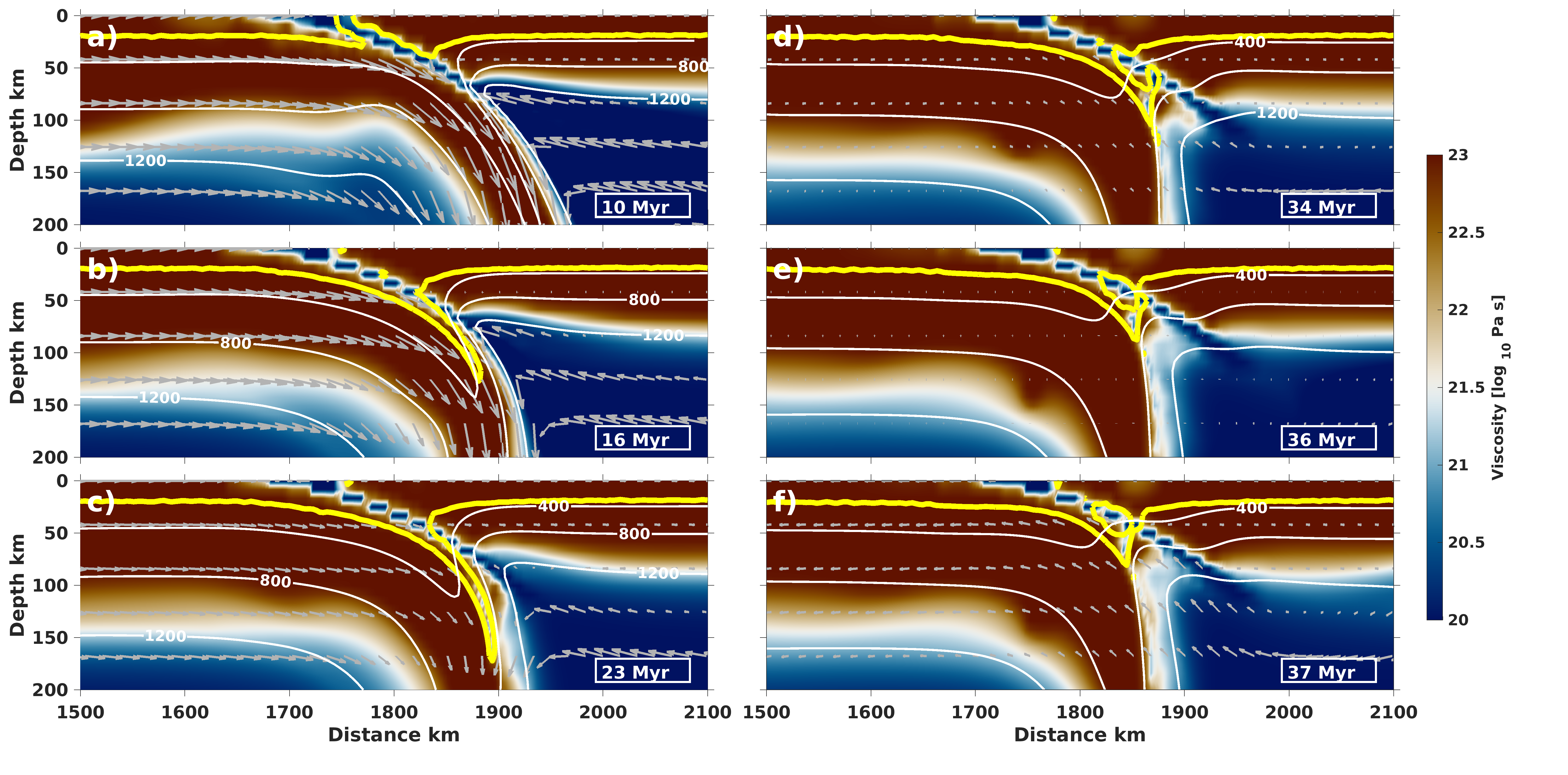


Figure S1: As **buoyancy test** for **subduction channel crustal exhumation,** we halved the crustal thickness and doubled the density contrast (70 Myrs slab and ∆ρ = 1418 kg/m^3^) to achieve the same total crustal buoyancy as in the default calculation (see main text for further explanation). Viscosity (colours), temperature (white contours, in ^o^C) and velocity (grey arrows). Yellow contour outlines the continental crust. These models have the same characteristic dynamics as the one presented in this paper and show that the total crustal buoyancy controls the dynamics .


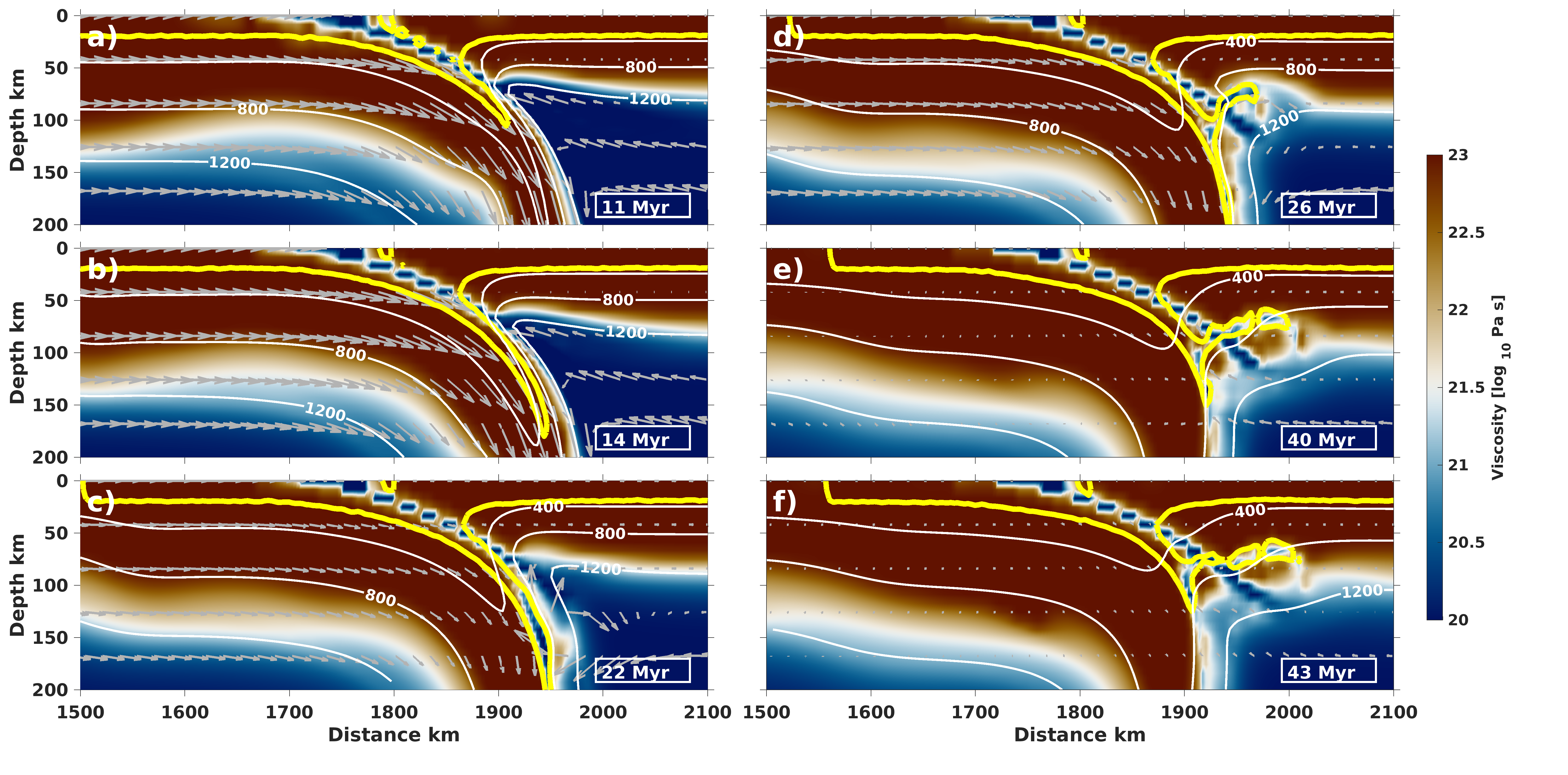


Figure S2: Viscosity (colours), temperature (white contours, in ^o^C) and velocity (grey arrows) of the **buoyancy test** for **underplating** (70 Myrs slab and ∆ρ = 992 kg/m^3^). Yellow contour outlines the continental crust. We halved the crustal thickness and doubled the density contrast to achieve the same total crustal buoyancy. These models have the same characteristic dynamics, as the one presented in this paper and show that the total crustal buoyancy controls the dynamics. There is less underplated material due to less deeply subducted continental crust.


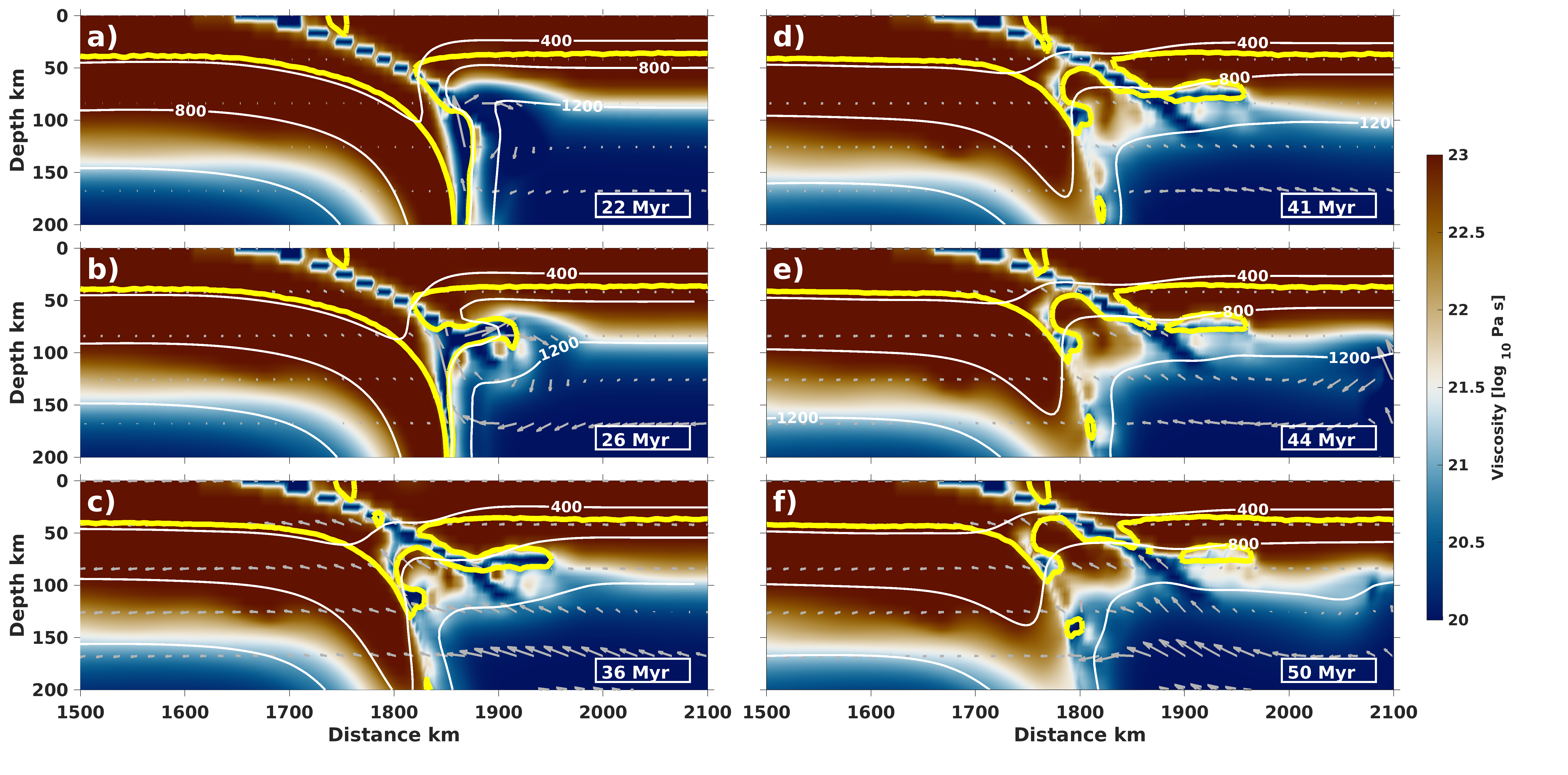


Figure S3: Viscosity (colours), temperature (white contours, in ^o^C) and velocity (grey arrows) of a **transitional model** between **subduction channel crustal exhumation** and **underplating** (50 Myrs slab and ∆ρ = 496 kg/m^3^). Yellow contour outlines the continental crust. The crust dominantly exhumes through the subduction channel, but is also partially place below the overriding plate. Note that convergence ceases before exhumation, unlike the ongoing convergence during underplating (Figure 5).


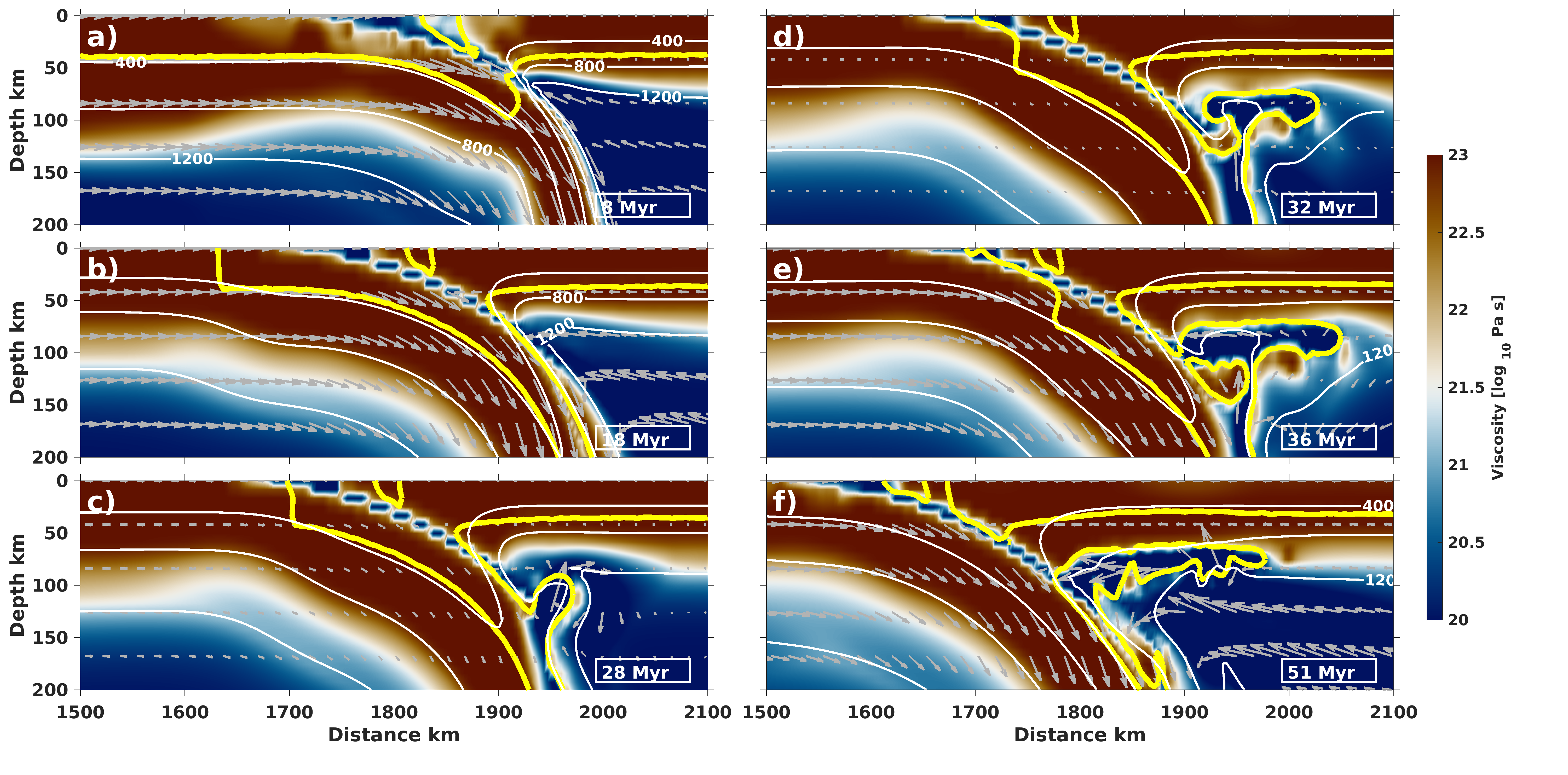


Figure S4: Viscosity (colours), temperature (white contours, in ^o^C) and velocity (grey arrows) of a **whole crustal subduction model** (70 Myrs slab and ∆ρ = 280 kg/m^3^). Yellow contour outlines the continental crust. During collision convergence rates are merely reduced, not ceased. This leads to subduction of all continental crust which partially underplates and is partially subducted deeper into the mantle.

Figure S5: An example PT-diagram of the stability field for upper crust composition at 2 wt% hydration with composition from Rudnick & Gao, (2003) and the solidus is based on Holland & Powell, (1998). The database consists of several diagrams with hydration varying from 0 wt% to 5 wt% with 0.05 wt% increments. See supplementary material of Freeburn et al., (2017) for details.

Figure S6: An example PT-diagram of the stability field for lower crust composition at 2 wt% hydration with composition from Rudnick & Gao, (2003) and the solidus is based on Holland & Powell, (1998). The database consist of several diagrams with hydration varying from 0 wt% to 5 wt% with 0.05 wt% increments. See supplementary material of Freeburn et al., (2017) for details.


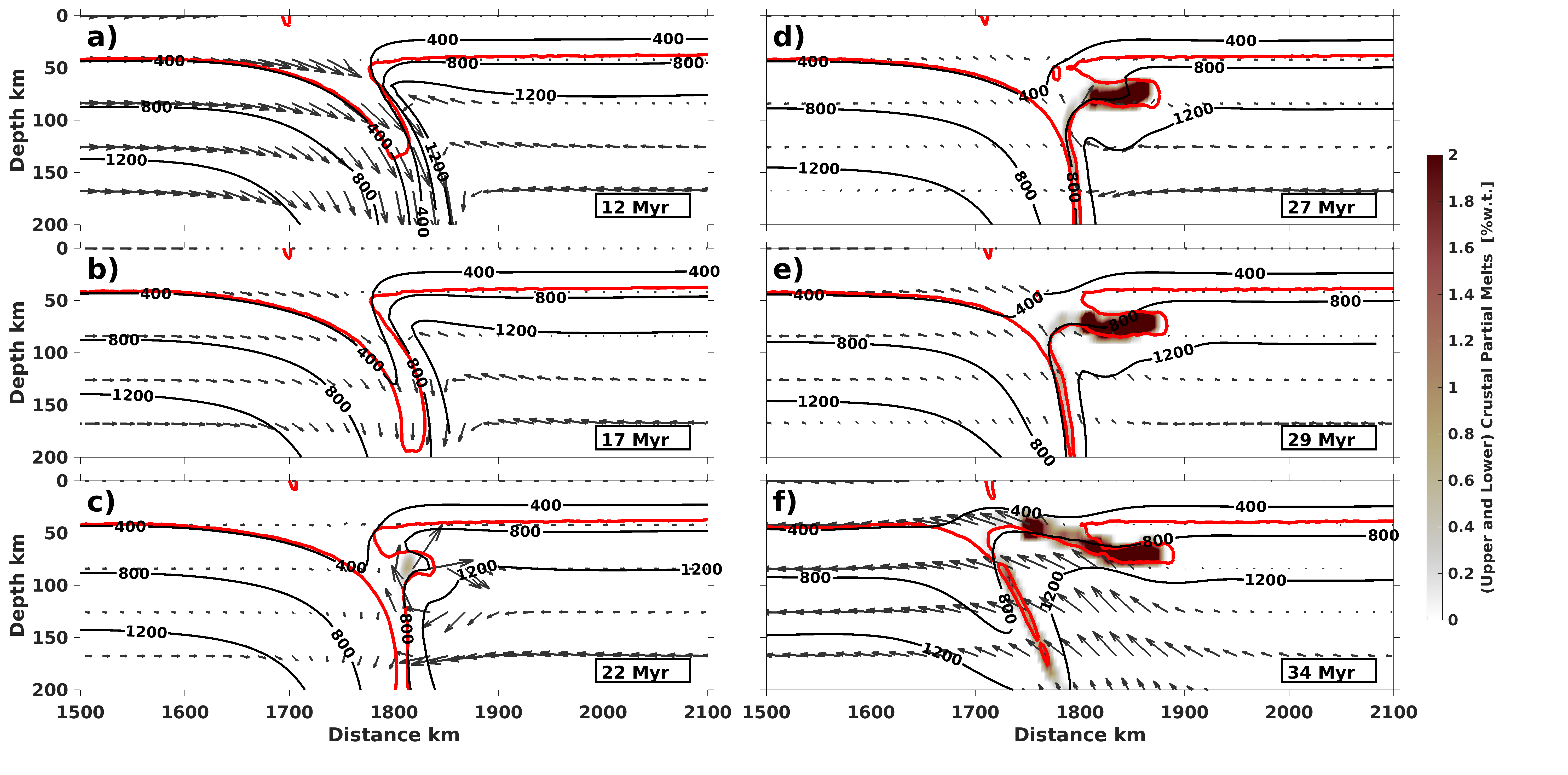


Figure S7: Evolution of crustal melting during **subduction channel crustal exhumation** (70 Myrs slab and ∆ρ = 709 kg/m^3^). Red contour outlines the continental crust.


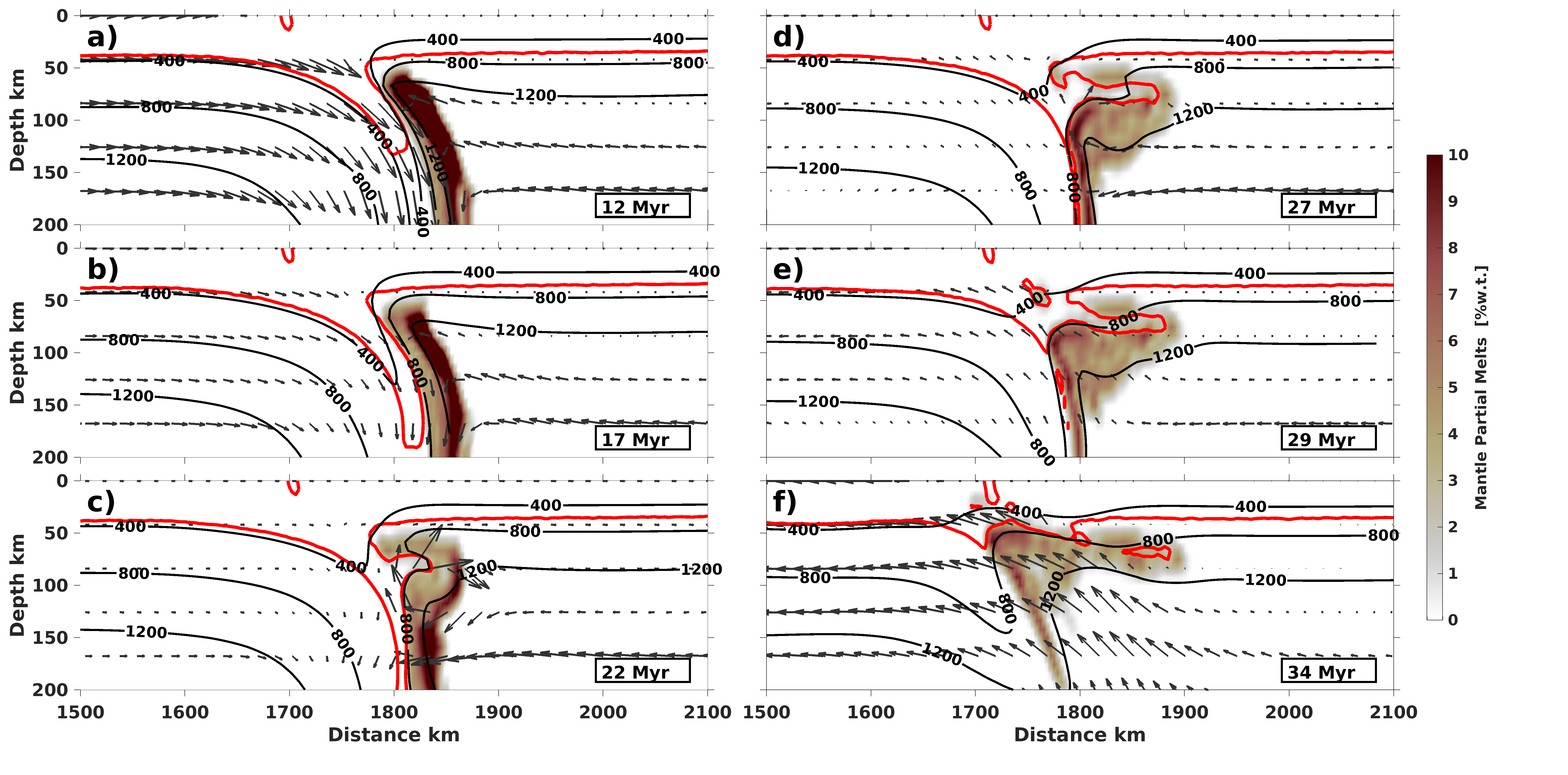


Figure S8: Evolution of mantle melting during **subduction channel crustal exhumation** (70 Myrs slab and ∆ρ = 709 kg/m^3^). Red contour outlines the continental crust.


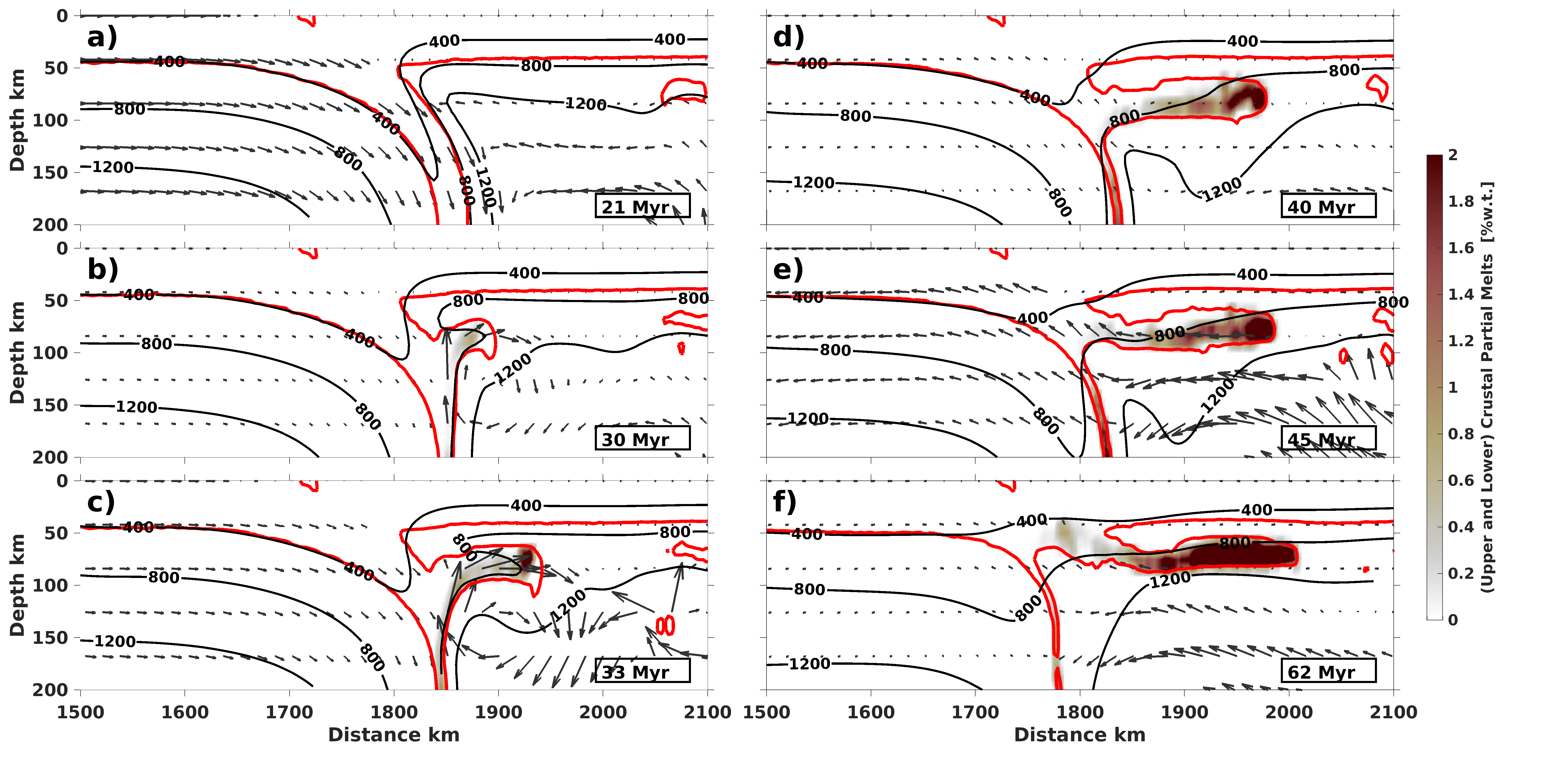


Figure S9: Evolution of crustal melting during **underplating** (70 Myrs slab and ∆ρ = 496 kg/m^3^). Red contour outlines the continental crust.


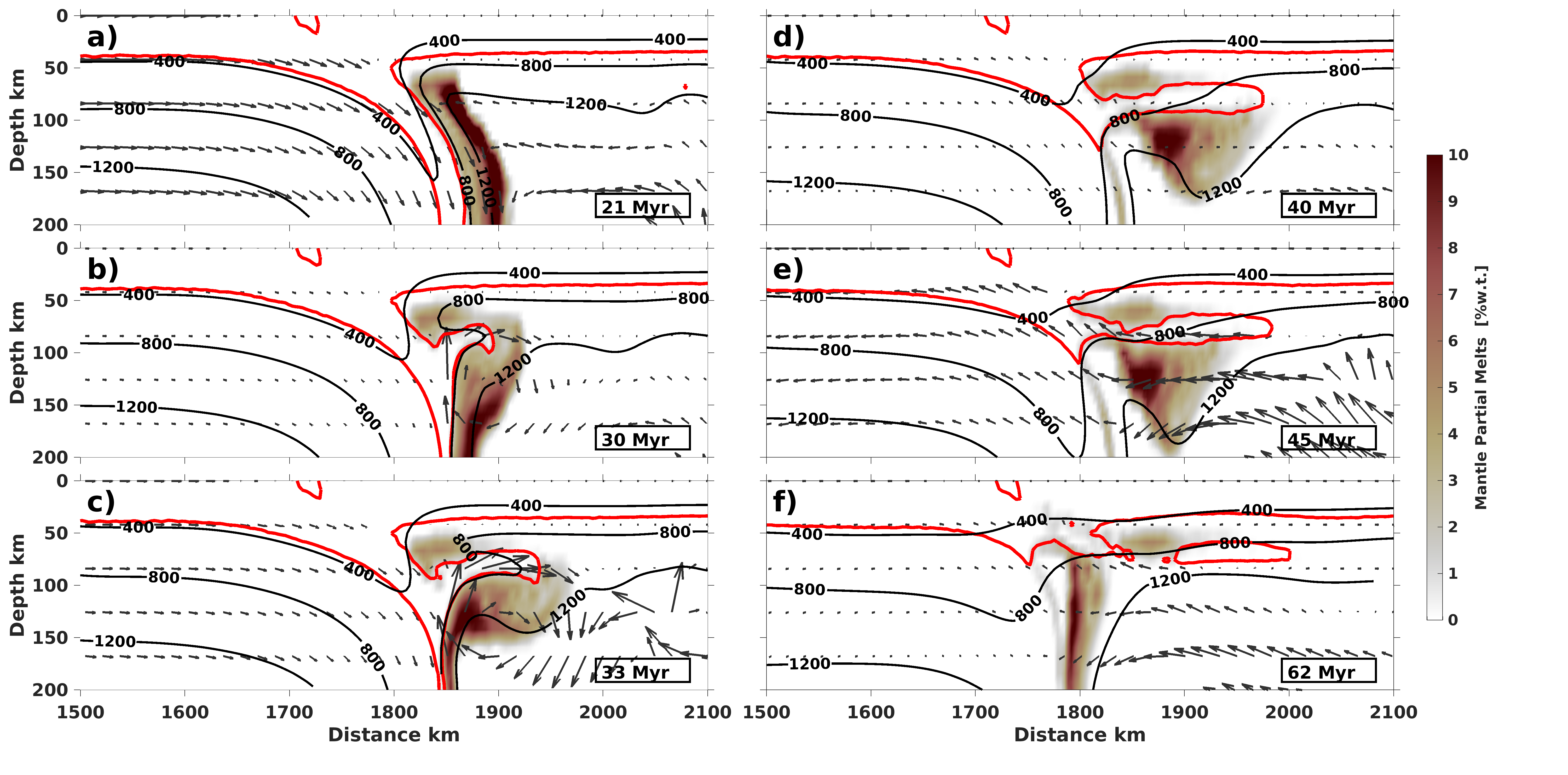


Figure S10: Evolution of mantle melting during **underplating** (70 Myrs slab and ∆ρ = 496 kg/m^3^). Red contour outlines the continental crust.
